# Repetitive Element-Mediated Recombination as a Mechanism for New Gene Origination in *Drosophila*

**DOI:** 10.1371/journal.pgen.0040003

**Published:** 2008-01-18

**Authors:** Shuang Yang, J. Roman Arguello, Xin Li, Yun Ding, Qi Zhou, Ying Chen, Yue Zhang, Ruoping Zhao, Frédéric Brunet, Lixin Peng, Manyuan Long, Wen Wang

**Affiliations:** 1 Chinese Academy of Sciences (CAS)—Max Planck Junior Research Group, Key Laboratory of Cellular and Molecular Evolution, Kunming Institute of Zoology, Chinese Academy of Sciences, Kunming, Yunnan, China; 2 Graduate School of Chinese Academy Sciences, Beijing, China; 3 Committee on Evolutionary Biology, The University of Chicago, Chicago, Illinois, United States of America; 4 Department of Ecology and Evolution, The University of Chicago, Chicago, Illinois, United States of America; Stowers Institute for Medical Research, United States of America

## Abstract

Previous studies of repetitive elements (REs) have implicated a mechanistic role in generating new chimerical genes. Such examples are consistent with the classic model for exon shuffling, which relies on non-homologous recombination. However, recent data for chromosomal aberrations in model organisms suggest that ectopic homology-dependent recombination may also be important. Lack of a dataset comprising experimentally verified young duplicates has hampered an effective examination of these models as well as an investigation of sequence features that mediate the rearrangements. Here we use ∼7,000 cDNA probes (∼112,000 primary images) to screen eight species within the Drosophila melanogaster subgroup and identify 17 duplicates that were generated through ectopic recombination within the last 12 mys. Most of these are functional and have evolved divergent expression patterns and novel chimeric structures. Examination of their flanking sequences revealed an excess of repetitive sequences, with the majority belonging to the transposable element DNAREP1 family, associated with the new genes. Our dataset strongly suggests an important role for REs in the generation of chimeric genes within these species.

## Author Summary

In numerous organisms, many new genes have been found to originate through dispersed gene duplication and exon/domain shuffling. What recombination mechanisms were involved in the duplication and the shuffling processes? Lack of the intermediate products of recombination that share adequate sequence identity between homologous sequences, or the parental sequences from which the new genes were derived, often makes answering these questions difficult. We identified a number of young genes that originated in recently diverged branches in the evolutionary tree of the eight Drosophila melanogaster subgroup species, by using fluorescence in situ hybridization with polytene chromosomes. We analyzed the genomic regions surrounding 17 new dispersed duplicate genes and observed that most of these genes are flanked by repetitive elements (REs), including a large and diverged transposable element family, DNAREP1. Several copies of these REs are kept in both new and parental gene regions, and their degeneration is correlated with the increasing ages of the identified new genes. These data suggest that REs mediate the recombination responsible for the new gene origination.

## Introduction

Gene duplication followed by the acquisition of novel molecular function is a fundamental process underlying biological diversity. It has been theoretically and empirically demonstrated that functionally distinct duplicates are capable of evolving through a neofunctionalization process in which there is an accumulation of mutations in a redundant copy of a preexisting gene [1–3]. In addition, there is mounting evidence for the rapid generation of new genes through the recombination of preexisting exons and functional domains. This latter process does not exclude, and in fact often relies on, the duplication of the loci involved [4,5]. Excluding chimeric genes formed through retroposition [6–8], more than three hundred gene families are believed to have originated through exon shuffling [9]. Most of these gene families have introns, suggesting that DNA level recombination was involved (DLR; DLR as opposed to a retroposition event involving an RNA intermediate).

Since its initial proposal [10], the genetic mechanisms involved in the formation of chimeric genes through exon shuffling have largely remained a mystery. The classic model states that nonhomologous recombination (NHR) brings together exons or domains from ectopic positions [10]. Experimental evidence for the role of NHR has been gained through transfection experiments [11,12] and through surveys of rearrangement hotspots which are often disease-associated [13–15]. Breakpoint analyses on these datasets revealed little or no sequence identity between the loci recombined, supporting a NHR model. While these experiments show such a model is possible for exon shuffling, it remains an open question how frequently such processes in non-artificial systems, and over evolutionary time, will contribute to the formation of fixed chimeric genes.

Another potential NHR mechanism that can mediate nonhomologous recombination is through the activity of transposable elements (TEs). If a TE is capable of mobilizing adjacent sequence, novel junctions that share no sequence identity could be generated [16]. The capacity for such events has been documented with the imprecise excision of well studied TEs such as P elements [17] as well as in plant pack-MULE and Helitron TEs [18–20]. These investigations implicate a role for TEs in the generation of chimeric genes. Whether these shuffled products are under functional constraint remains an interesting question.

Alternatively, non-allelic homologous recombination (NAHR) between ectopic sequences can lead to the formation of chimeric genes. Recently, a surge of evidence has begun to demonstrate the importance of NAHR to genomic architecture, especially in primates [21–26]. Intriguingly, several studies have reported on a limited number of chimeric gene structures, some of which appear functional and nondeleterious, but most remain putative [24,27]. Focus has primarily been placed on NAHR's role in human disease [26]. However, given that NAHR appears to be a common mutational mechanism, a new hypothesis for exon shuffling has been motivated: Despite the frequently deleterious effects, NAHR is capable of making a contribution to the origin of new chimeric genes as an exon shuffling mechanism [24,25,28,29].

A difficulty in investigating the relative contributions of these mechanisms to the formation of chimeric genes is that most of the available examples are evolutionarily ancient [9]. These genes provide few clues for understanding the recombination mechanisms that generated their initial structures because the sequence features, especially those non-constrained sequence traits, that may have fostered their formations have likely been lost (the half life is 120 mys for mammals and 10 mys in *Drosophila* [30]). While sequence analyses of ancient chimeric genes provide little mechanistic insight, a sample of young chimeric genes that potentially retain these sequence features may. A second difficulty arises from the limited number of young chimeric genes that are thought to have arisen by DLR. While several case studies exist, evolutionary analyses demonstrating that the new chimeras are functional are largely lacking [24,27,31].

Here we report on a large-scale experimental genomic screen for young chimeric genes generated by DLR within the D. melanogaster subgroup. We utilized an integrated approach based on fluorescent *in situ* hybridizations (FISH), Southern hybridizations, expression and transcript experiments, BLAST queries, and evolutionary analyses. This approach allowed us to focus on dispersed duplication events, ignoring tandem duplications. Consequently, the total number of chimeric formations are likely larger than the total we report on here. Nonetheless, our results show that, rather than providing redundant copies, dispersed duplication events via DLR have generated new chimeric structures at a high frequency. Interestingly, none of these chimeric structures involved two or more genic sequences; all chimeric regions were formed from the fusion of the duplicated loci and intergenic sequences. Furthermore, we provide strong evidence that REs, in particular the TE family DNAREP1, are a major mediator of these events. Finally, using multiple well-established methods [6,7,32–34], we demonstrate that most of these new chimeric genes are functional.

## Results/Discussion

Two cDNA unigene libraries from D. melanogaster comprised of ∼7,000 cDNA probes were used for cFISH experiments over all tested species. Each hybridization generated at least two images for each species. In total, our experiment produced ∼112,000 primary images. Including those probes that gave weak or paradoxical signals, the *Drosophila* Gene Collection (DGC) library version 1.0 set resulted in 266 candidates. The unigene library included 1,000 cDNA probes, most of which were included in the DGC 1.0 library. From this set, 5 new genes, *jingwei* [33], *Hun* [32], *sphinx* [34], *monkey-king* [35], and *Dntf-2r* [36] have previously been described.

**Figure 1:**
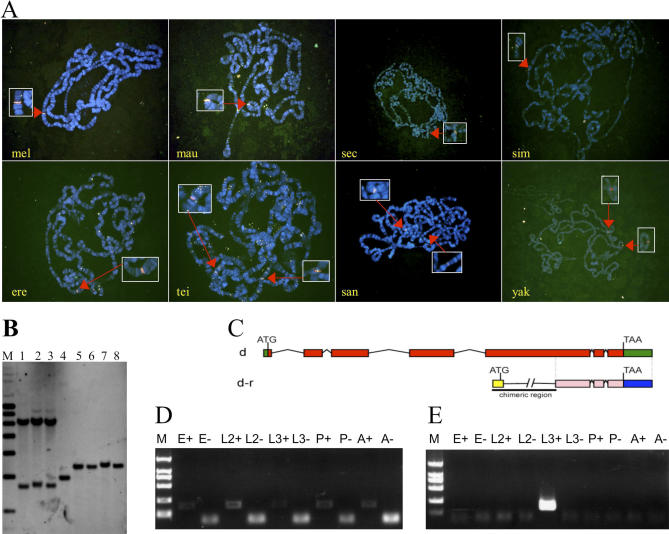
An Example Illustrating the Detection of New Genes (A) The probe LD47348 (CG10595) detected two signals in the clade of *D. yakuba-santomea-teissieri* while only detecting one signal in other species. The new additional signal suggests a new gene candidate. (B) Southern hybridization results further confirm the extra copy in the *D. yakuba-santomea-teissieri* clade (M is 1-kb extension marker [Invitrogen]). Lanes 1–8 correspond to Xho I digested DNAs of D. yakuba, D. teissieri, *D. santomea*, D. erecta, D. melanogaster, D. simulans, *D. mauritiana*, and *D. sechellia*, respectively). (C) Cartoon figure displaying the gene structures of the parental gene (d, or CG10595) and the new duplicate (d-r). The duplicated region is indicated by vertical dash lines. d-r recruited one upstream exon as indicated by yellow box. (D) Expression patterns of the parental gene. (E) expression patterns of the new gene d-r revealed by one round of RT-PCR and a second round of nested PCR (M indicates DL2000 DNA molecular marker (Takara); E+, E−, L2+, L2−, L3+, L3−, P+, P−, A+, and A− correspond to positive and negative reactions for embryos, second instar larvae, third instar larvae, pupae, and adults, respectively). From these gels, it is clear that d-r is only expressed in the third instar larvae while the parental copy is expressed ubiquitously. All the bands in the negative control lanes are primer dimer bands. E+ and L3+ are weak but clearly visible.

**Figure 2:**
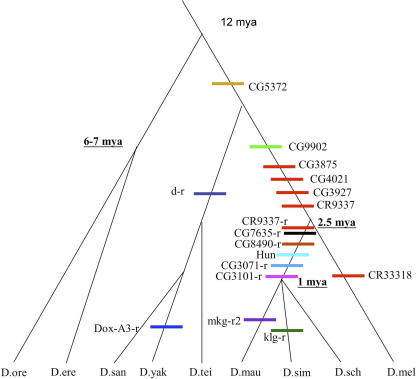
The Phylogenetic Distribution of the 17 New DLR Duplicates Identified in This Study The species phylogeny and time scale are from [58]. Different color bars show different gene families. The kep1 gene family has six new duplicates (indicated by red bars).

To exclude false positives from the 266 candidates, we carried out Southern hybridizations and conducted BLAST searches against the available genome sequences of D. simulans (droSim1)*, D. yakuba* (droYak1)*, D. sechellia* (droSec1)*, D. melanogaster* (dm2) and *D. erecta* (droEre1) (http://genome.ucsc.edu) (Figure 1). The Southern and BLAST analyses confirmed 17 young duplicates generated through DLR (Table 1; Figure 2). The genomic sequences of all 17 dispersed duplicates contain the intron(s) and/or non-coding flanking sequences that exist in their parental copies, suggesting that the new genes originated through DLR. In addition, we also identified ten new copies of retrogenes and 53 young copies of REs including retroelements and other repetitive sequences. In this report, we have focused on the 17 dispersed duplicates and investigate possible DLR mechanisms that generate dispersed duplications.

**Table 1:**
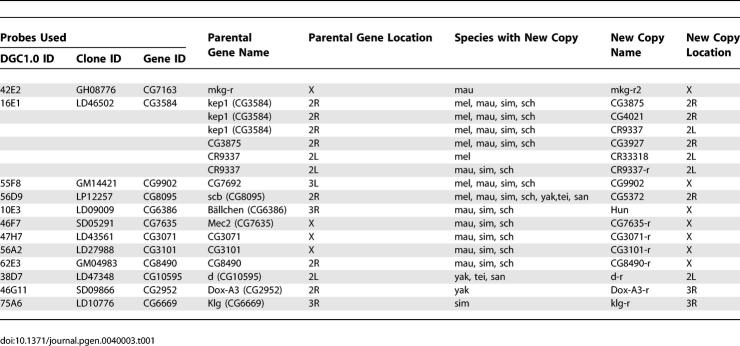
List of the Young Duplicates Identified and their Parental Loci

Interestingly, the *kep1* gene family has six new duplicates that have been dispersed to different chromosomal locations, while the other 11 gene families have only a single new duplicate (Table 1). Thirteen of these duplications are intrachromosomal, and 4 are interchromosomal (Table 1). Two putative pseudogenes exist in this list: CR33318 and CR9337. CR33318 is found only in D. melanogaster, however CR9337 has a disrupted reading frame in D. melanogaster but is intact in D. sechellia and *D. simulans*. Mapping these results onto the species tree reveal an age <8 mys for almost all these origination events except the 12-my-old CG5372 (Figure 2).

Excluding the two putative pseudogenes (CR33318 and CR9337) paralog-specific reverse transcriptase (RT-PCR) experiments detected transcripts for all paralogs. Twelve out of these 15 duplicates display differential expression patterns from their parental copies in development and/or sex (Table S1). These observations indicate that most of the new genes have evolved divergent expression patterns, and that generally the patterns are more restricted.

**Figure 3:**
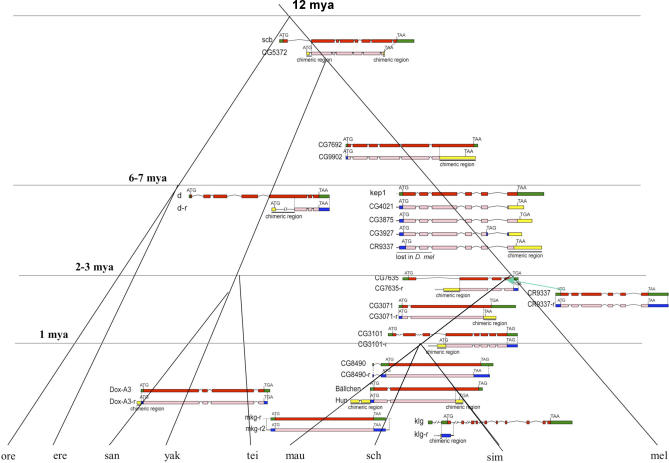
The Gene Structures of 16 New Duplicates Mapped on the Species Phylogeny CR33318 is not shown because it is a truncated copy without detectable expression and has frame shift mutations. Duplicated regions are indicated with vertical dash lines. Horizontal dash lines in CG7635-r, CG3101-r, d-r, and klg-r indicate that we only obtained partial coding regions with RT-PCR and longer coding regions may exist outward. Boxes are exon regions and lines indicate introns. Yellow boxes indicate recruited chimeric regions, green boxes indicate parental loci UTRs, and blue boxes indicate duplicate loci UTRs. Positions of start and stop codons are marked.

To examine whether the new duplicates have evolved chimeric gene structures, we utilized previously reported cDNA sequences, RACE, or RT-PCR based on computationally predicted structures (Materials and Methods). Among the 17 new genes, 13 were found to have evolved chimeric gene sequences through the recruitment of flanking sequence near the insertion site or as the result of extensive deletions (CG5372, CG9902, CG4021, CG3875, CG3927, CR9337, CG7635-r, CG3101-r, CG3071-r, *d-r*, Dox-A3-r, *Hun*, and *klg-r*; Figure 3). Among these chimeric genes, 11 can encode chimeric proteins (CG5372, CG9902, CG4021, CG3875, CG3927, CR9337, CG7635-r, CG3101-r, CG3071-r, *Hun* and *d-r*). For example, *d-r* and CG9902 have both recruited novel coding regions following their duplications, and possibly in conjunction with their deletions events that followed (Figure 3). These observations reveal that the majority of young duplicated genes have evolved chimeric gene structures. In addition, it is notable that the chimeric genes that we have detected involve only the duplicated loci and integenic sequences. This suggests that for dispersed duplication events, the formation of chimeric genes by recombining two or more genic sequences may be relatively rare.

To test for functional constraint, we conducted substitution analyses by estimating the Ka/Ks ratio for both paralogous and orthologous comparisons. For the paralogous comparisons, our conservative null hypothesis was that the parental genes are under strong functional constraint with the new copy subject to no constraint (a pseudogene). These estimates suggest that most of the genes are under functional constraint: Ka/Ks values are lower than 0.5 for 8 genes, lower than 1 but higher than 0.5 for 5 genes, and ∼1 for 2 genes (Table S2). Furthermore, analyses of the functional domains for these genes (Materials and Methods), revealed that almost all genes have Ka/Ks ratios lower than or close to 0.5 (Table S2). For orthologous comparisons, the null hypothesis was that the new copies are pseudogenes (Ka/Ks = 1). The results were similar, showing that Ka/Ks ratios are significantly less than 1 for most genes except CG3071-r (Ka/Ks = 2.3091) and CG8490-r (Ka/Ks = 1.2230), indicating the possibility that positive selection may be acting on these two (Table S3). The statistical tests of the null hypothesis of neutrality [1] in the paralogous and orthologous comparisons reveal that most of these new genes are under significant functional constraint over the tested coding sequences. These complementary analyses of expression, gene structure, and nucleotide substitution suggest that all 15 new genes are functional and that many of these have undergone neofunctionalization by evolving new gene structures with new expression patterns.

The classical models of gene duplication assume a completely redundant (in sequence and function) duplicate copy [1,2]. In these models the most likely outcome is that one copy will become non-functionalized, with a low probability that one or the other becomes neofunctionalized or subfunctionalized through subsequent mutations [37,38]. However, our results show that the majority of new duplicates generated through DLR in *Drosophila* are not structurally, and are thus unlikely to be functionally, identical to their parental copies. It is also a general result that DLR is an important mechanism for the generation of dispersed genes with novel functions, adding to other potential mechanisms [39]. Interestingly, Katju and Lynch [40] have recently found that many new duplicates in C. elegans have unique exons in one or both members of a duplicate pair. Consistent with our observations, these latter cases are also likely DLR-derived duplicates that have recruited new gene fragments and have evolved stable chimeric structures.

Having established that 15 of these new duplicates are likely functional, with many having chimeric structures, we then investigated the mutational mechanisms that generated them. Data, largely originating from detailed sequence analyses of human disease-related loci, have shown correlations between structural variation and REs, most notably *Alu* elements in primate genomes [13,22,23,25,28,41]. Though a causal relationship between the repetitive elements and segmental duplications is difficult to establish, several studies have argued for their causative role in genomic rearrangements through NAHR. Based in part on these findings, we were interested in whether there was evidence for repetitive sequence surrounding these duplicated regions.

We identified both 5′ and 3′ breakpoints for each young duplicate by comparing genomic sequences of each of these new gene duplicates with its parental copy (Table 2). Interestingly, we observed REs at or near the breakpoints for 10 out of the 17 duplicates (including the 2 duplicates that are likely pseudogenes) (Table 2; Figure S1). These REs consist of 7 TEs, 2 satellite sequences, and 1 simple repeat. They are associated with the new genes that are in different genomic locations, suggesting independent events. Furthermore, all TEs belong to the DNAREP1 family, the largest TE family in *Drosophila* which has very diverged members [42,43].

**Table 2:**
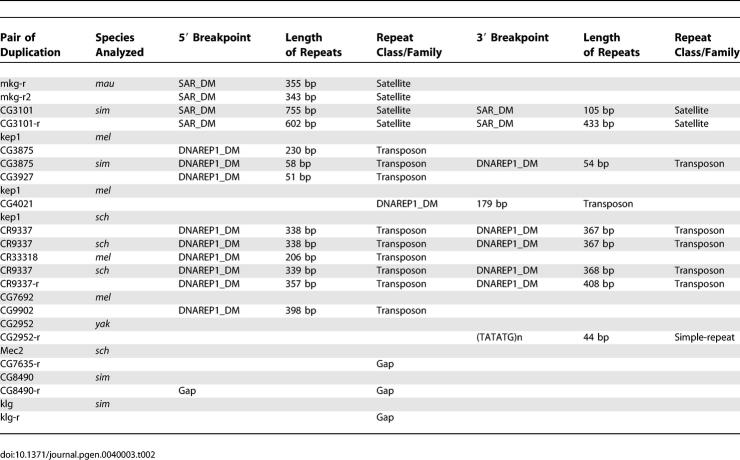
Repetitive Elements at the Breakpoints of Duplicate Pairs

**Figure 4:**
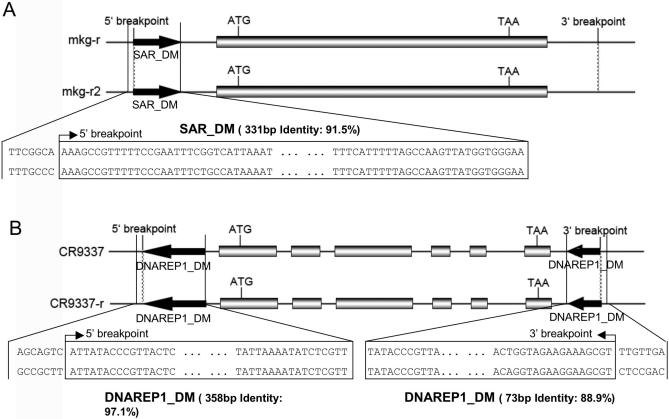
Two Examples of New Genes with Repetitive Sequences at the Breakpoints (A) Shows a satellite DNA sequence (SAR) located at the 5′ breakpoints of mkg-r2 and its parental gene mkg-r. (B) Shows the existence of a transposon (DNAREP1-DM) at all the four breakpoints of a CR9337 duplicate pair.

Among these 10 pairs associated with REs, 5 have shared repeats at or near the breakpoints of both the parental and the new duplicate copies (Table 2). For these 5 paralog pairs, 4 (CG3875-CG3927, mkgr-mkgr2, CG3101-CG3101-r and CR9337-CR9337-r) maintain very high sequence identity over the flanking elements; the remaining CR9337-CR33318 pair, though both harboring DNAREP1 sequence at their 5′ ends, provides a weak alignment. The other five paralog pairs contain a repetitive element at the breakpoint of one copy (Table 2; 2 examples with highly similar TEs shown in Figure 4). In addition, *klg-r*, CG7635-r and CG8490-r (not included in the ten above) were found next to sequencing gaps in the genomic databases (Table 2), and resequencing these regions resulted in sequence profiles characteristic of repetitive sequences (data not shown). If these are included, the majority of new duplicates (13/17, 76.5%) are associated with repetitive elements.

**Figure 5:**
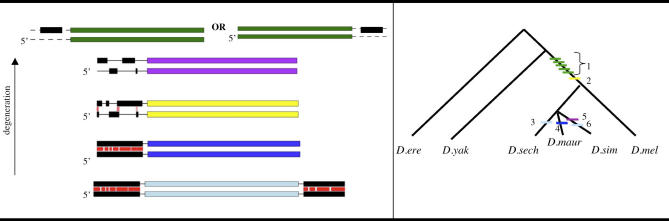
A Simplified Schematic of the Repetitive Sequence Flanking New Genes and Their Distribution over the D. melanogaster Subgroup Phylogeny Left panel displays the varying degrees of identity and degeneration between flanking regions of paralogs, with the right panel displaying the branches in which they are found; 1: (CG2952:CG2952-r), (kep1: CG4021), (kep1:CG9337), (CG9902:CG7692), (kep1:CG3875); 2: (CG3875-CG3927); 3: (CG9337-CG9337-r); 4: (mkgr-mkgr2); 5: (CR9337-CR33318); 6: (CG3101-CG3101-r). The red blocks in the left panel indicate alignable regions of the TEs and other repeat sequences. The black boxes represent sequences of TEs and other repeats; fragmented black boxes represent RE fragments. The long boxes in various colors represent the identified new genes. See also Figure S1, for the alignments.

Four lines of evidence indicate that this association has not been observed by chance. The first is based on orthology assignments available from current genome databases, indicating that all ten in our set are euchromatic and not on the 4^th^ chromosome. High-resolution analyses of D. melanogaster TEs have verified that the paracentromeric regions of the major chromosome arms and chromosome 4 harbor the highest densities of TEs [44]. Second, simulations show that the probability that the number of genes flanked by TEs ≥7 given the sample size of seven genes (with 14 breakpoints) is low (*p* < 0.05) given a TE-free region (TFR) of ∼15 kb or larger (Figure S2; Materials and Methods). Despite TE differences between species, 15 kb is less than half the mean TFR found in D. melanogaster [44]. Given that the TEs in our dataset are comprised primarily of DNAREP1 family members, the distance is even greater. Furthermore, the probability that both paralogs contain the same TE sequence in their flanking regions, as three (and possibly four) do in our dataset, is much lower (Table 2; Figure S1). Finally, our data reveal a gradation of degeneration in the TEs and other REs with the ages of the gene duplicates that the repeats flank (Figure 5). This gradation is consistent with observed degeneration rate of functionless elements in *Drosophila* [30], as well as any potential internal deletions that could be part of a self-regulation system as seen in D. melanogaster TEs [45].

The striking association with REs provides evidence for the relationship between RE sequences and genomic rearrangements leading to novel functions. This relationship differs from previous reports of TE themselves becoming part of a novel transcript in D. melanogaster [46,47]. Instead, our dataset supports a model whereby REs are mediating the recombination of flanking sequences to form chimeric products that do not include RE sequence. The precise mechanism defining “RE-mediation” would likely be NAHR or the mobilization of flanking sequence through the activity of the DNAREP1 transposons. Recent studies of DNAREP1 elements suggest a burst of activity occurred just prior to or during the formation of the D. melanogaster subgroup, followed by nearly complete inactivation ∼5–10 mya [42]. Interestingly, there is evidence of a very recent revival of activity in the D. yakuba lineage [43]. If these estimates on inactivity are correct, NAHR would be the most likely mechanism generating the rearrangement in our dataset. This possibility is also supported by the identified non-mobile repeat sequences that are associated with the new chimeric genes (Table 2). However, if DNAREP1 has been active in the D. melanogaster subgroup for a longer period than reported, as implicated by the observation in D. yakuba [43], and if this class of TEs does in fact mobilize flanking DNA, a combination of mechanisms is possible.

Alternatively, the REs flanking the new duplicates could be the result of larger duplications that included the REs (segmental duplication), rather than the REs mobilizing the region. However, we would expect that under this hypothesis we would see longer stretches of identity outside REs. Inspecting the flanking regions of our dataset indicate that identity is lost in close proximity with the repetitive sequences. A second alternative hypothesis is that the repetitive sequence presents a preferential site for strand breakage. Similar suggestions have been made for *Alu*, satellite repeats, and other sequence demonstrating fragility [23,31,48]. If imperfect repair were to follow strand breakage, this too would be akin to a nonhomologous end-joining event and would support the classical view of exon-shuffling. Further experimental work is needed to address this possibility.

Our observation that there is an excess of repetitive elements around dispersed functional duplicates is of general importance in light of advancements in identifying copy number variation in other model organisms, and the increased recognition for the role of repetitive sequences in shaping chromosomal architecture [14,22–26,31,49,50]. Despite these advancements, little is known about the potential non-deleterious outcomes that such rearrangements may present. Our work helps fill this void by providing an extensive chimeric gene dataset that is supported by experiments that test for functionality.

Evidence from previous case studies has indicated that once a duplicate has been generated the recruitment of exons and/or flanking gDNA is a heterogeneous process [32,51]. Within our dataset, we also observe this. The first instance is the direct recruitment of genomic DNA flanking the insertion site of the new copy. Eight new genes, representing eight gene families (CG9902, CG5372, *Hun*, CG7635-r, CG3701-r, *d-r*, *Dox-A3-r* and *klg-r*), were created this way. The second involves dramatic mutations within the new duplicates. In the *kep1* family, numerous deletions in the duplicated 3′ regions have resulted in varying peptide sequences in the C terminal (Figure 3). In CG8490-r, both the start and the stop codons have been shifted, resulting in different peptides at both N and C termini. Finally, CG3101-r has recruited part of its previous intron 3, which becomes the 5′ UTR and a short stretch of protein-coding sequence in the new duplicate gene.

We have used ∼7,000 cDNA probes to screen new gene duplicate copies. The estimated number of genes in the genome is ∼14,000. The total number of new gene duplicates can be estimated as 17/7,000 × 14,000 = 34, over an evolutionary time equal to ∼20 mys (the sum of the branch lengths of the D. melanogaster subgroup). Thus, on average, the origination rate is 34/20 = 1.7 per mys per genome, or 0.121 × 10^−9^ per year per gene. We note that, because our method ignores tandem duplicates, and because our FISH probes were all based on D. melanogaster sequence, this is an underestimate. However, this rate is an order of magnitude higher than the gene duplication rate estimated in yeast [52] but still 30 times lower than a previous estimate that were based on the assumption of a molecular clock [53]. Our estimate may not be inconsistent with previous estimates [53] because our focus was much narrower, investigating DLR events only.

Only two new duplicates (*d-r* and *Dox-A3-r*) in the *yakuba-santomea-teissieri* lineage (*yakuba* lineage) were observed, while 5 new duplicates were detected between the common ancestor of *melanogaster* and *yakuba* and the common ancestor of the *melanogaster* complex (Figure 2). In addition, we did not detect any new duplicate in D. erecta. This may be a technical result attributable to the difficulty of hybridization with D. erecta polytene chromosomes, or sequence divergence relative to our probes. Alternatively, the putative inconsistent duplication rate may be associated with episodic activities of transposons or repetitive sequences. For example, the transposon DNAREP1 members were associated with 5 new duplicates in the *kep1* gene family and CG9902. As noted above, it has been suggested that there was an active episode of DNAREP1 before the D. melanogaster lineage separated from the D. yakuba lineage and then again within the D. yakuba lineage [42,43].

Previous investigations have revealed several important roles for REs in the generation of evolutionary novelties including the donation of their own sequences into protein coding regions [46,47,54,55], retrotransposing and recruiting novel gene sequence [5], increasing genic diversity in the maize genome by the helitron-like transposons [56], potentially providing greater overall genome plasticity [16,57], and elevating expression of a nearby insecticide resistant gene [58,59]. The observation reported here further demonstrates a mechanistic role for REs in mediating the origins of new genes by facilitating gene recombination. The precise mechanism for this recombination is unclear, but likely include NAHR, as implicated by both TEs and non-TE repetitive sequences being detected, and NHR as a consequence of transposon enzymatic activities [43]. However, the conventional NAHR model is much more likely between the homologous repeats that are located on the same chromosome [22]. Four of the 17 new genes identified are on different chromosomes from their parental genes. These four new genes may have been generated by a different homology-dependent recombination model that assumes a replication-dependent mechanism involving no crossover [22], the explicit model depicted in Figure 8.

## Materials and Methods

### Materials.

In order to screen for young chimeric genes systemically, we designed an experimental genomics approach using the D. melanogaster species subgroup as a comparative model system. This subgroup includes D. melanogaster (hereafter abbreviated as mel in presented tables and figures)*, D. simulans* (sim)*, D. mauritiana* (mau)*, D. sechellia* (sec)*, D. yakuba* (yak)*, D. teissieri* (tei)*, D. santomea* (san)*,* and *D. erecta* (ere). D. orena was excluded from analyses because of its unclear placement in the phylogeny. The phylogeny of this subgroup is well resolved [60,61] and the divergence times among these species provide a considerable range over which to detect the presence of young genes. The polytene chromosomes of the salivary gland of *Drosophila* allow detection of gene copy number using a fluorescent *in situ* hybridization (FISH) approach. Therefore, we can use cDNA probes to visualize FISH signals that are about 100 kb away from each other in the species of D. melanogaster subgroup, and count the signal number in each species [34].

### FISH and Southern hybridizations.

We carried out dual-color FISH on the polytene chromosome preparations of the aforementioned 8 species. Our probe sets comprised 5,928 full-length D. melanogaster cDNA clones from the Berkeley Drosophila Gene Collection (DGC) version 1.0 (http://www.fruitfly.org/DGC/index.html) and about 1,000 cDNA clones from an early Drosophila Unigene Library (Research Genetics).

Probes were labeled with digoxiginin (DIG) or biotin using PCR [34,62]. Polytene chromosomes from four species were simultaneously squashed on a slide and then hybridized with a pair of DIG and biotin labeled probes [34]. For a given probe, FISH is capable of resolving two signals across two adjacent polytene bands, which is equivalent to ∼100 kb in linear DNA sequence. As a result, all duplicates we report in this study have been involved in translocations; they are not tandem duplications. The probes that revealed extra signals in a particular lineage were subject to further confirmation using southern hybridization. Genomic DNAs of the eight species were extracted using the Puregene DNA isolation kit (Gentra Systems). DNAs digested with restriction enzymes were separated on agarose gels and transferred to nylon membranes (Roche Molecular Biochemicals) by Southern blotting. The DIG-labeled probes were hybridized to the membrane to further confirm the copy number in different species. In addition, homology searches were carried out for those new genes that fell within sequenced genomes (http://genome.ucsc.edu).

### Breakpoint analyses.

To identify breakpoints and examine the type of sequence surrounding them, the genomic sequences of each pair of duplicate and parental copy, along with 5′ and 3′ flanking sequences, were aligned using the bl2seq software with default settings [63]. The length of the 5′ and 3′ flanking sequences for each pair was chosen to ensure that it extends 1kb beyond the point where sequence identity disappears. Breakpoints of duplicates were determined as the last nucleotide showing sequence identity between parental and new copy. For a multiple-copy gene family, the parental copy was defined as the copy that has the highest similarity to the new copy. RepeatMasker (http://www.repeatmasker.org/) was used to identify whether there is repetitive sequence within a 100 bp window centered at each breakpoint.

### Substitution analyses.

To examine the evolutionary forces operating on the new duplicates, we calculated synonymous (Ks) and non-synonymous (Ka) divergence between all paralogs except for the pseudogene CR33318 (we included the putative pseudogene CR9337 and CR9337-r because they are still intact in the D. simulans complex). In addition, we also conducted substitution analyses between orthologous copies in different species. For 11 young duplicates we retrieved their orthologs from a second species's genome, and therefore also calculated Ka and Ks between the orthologous pairs. Estimates were obtained using MEGA 3.1 [64]. A Z-test implemented in MEGA 3.1 was used to test if Ka/Ks ratios deviate from the neutral expectation (Ka/Ks = 1). We tested functional constraint in the whole gene coding region and the functional domain separately. To define the functional domains, the coding sequences of genes were translated into the protein sequences. Then we performed rps-BLAST to detect whether the newly translated protein sequences have functional domains using a cutoff line E < 0.01 on NCBI website http://www.ncbi.nlm.nih.gov/Structure/cdd/wrpsb.cgi.

### RACE, RT-PCR, and gene structure analyses.

Our approach is capable of observing three kinds of new duplicates, (1) direct duplicates that still keep intron(s) or flanking non-coding sequences, (2) retroposed copies that have lost ancestral introns, and (3) copies that have no obvious sequence features identifying them as either created by retroposition or direct duplication. Tandem duplication can be resulted from either replication slippage or DLR, but the assumption is that those dispersed duplicates across long chromosome distance, or between chromosomes, have originated through DLR. In this study, we only considered direct dispersed duplicates that were derived through DLR. For each of these duplicate genes, we designed copy-specific RT-PCR primers. RT-PCR experiments were carried out using cDNA from 5 developmental stages: embryo, instar larva 2 (L2), instar larva 3 (L3), pupa and adult. Total RNA was extracted from these samples using RNAeasy Mini RNA extraction kit (Qiagen). To avoid contamination of genomic DNA, total RNA was treated with Dnase I (amplification grade, Invitrogen) prior to first strand synthesis. First strand cDNA was synthesized using Oligo-dT and SuperScript II Rnase H- reverse Transcriptase (Invitrogen). All RT-PCR products were sequenced for verification.

To establish the gene structures of the new genes, four types of data were used: (1) the draft genomes of D. simulans (droSim1)*, D. yakuba* (droYak1)*, D. sechellia* (droSec1)*, and D. erecta* (droEre1) (http://genome.ucsc.edu) were queried and provided addition verification and gDNA for primer design; (2) For those duplicates whose full length cDNAs are available in public databases (http://www.ncbi.nlm.nih.gov/Database/), we mapped the cDNA to their genomic positions if draft sequence was available; (3) For those duplicates without cDNA, and whose sequences have diverged enough to allow copy-specific primers, we carried out rapid amplification of cDNA ends (RACE); (4) For those duplicate pairs that are too similar to allow copy-specific primers, and for those that resulted in no RACE product (possibly due to low expression levels or long ends), we used the Softberry software [65] to obtain a tentative chimeric gene structure prediction. We then tested these predictions using RT-PCR.

### Chromosomal mapping.

To establish an approximate chromosomal position (interstitial or not) for each of these genes, we used the D. melanogaster genome as a reference. We carried out BLAST queries of the D. melanogaster genome using sequence flanking each of the genes. These flanking regions were then used to query available genome draft sequence (http://www.ncbi.nlm.nih.gov/Database/) in order to determine orthologous chromosomal regions. The cytological positions were then extracted using NCBI's MapView (www.ncbi.nlm.nih.gov/mapview/). Two new copies (CG7635 and klg), fell between sequence gaps. For these two we determined their approximate position based on our FISH images.

### TE association simulation.

To assess the significance of our observed association between TE sequences and the flanking regions of the paralogs, we carried out simulations based on the known frequencies of TEs in D. melanogaster [44]. The mean TE-free region (TFR) is 23,878, with a median of 1,992. The difference between the mean and the median results from the clustering of TEs within the pericentric regions and the fourth chromosome. However, the identified new genes are non-pericentromeric regions in which the density of TEs is much lower and there are few cases of non-random insertions to one particular locus. Therefore, we carried out simulations over a range of normally distributed TFRs in a conservative assumption of the 15 kb average. The length of each TE was normally distributed with a mean of 4 kb. The total length of simulated chromosomes was kept at ∼ 20 Mb. 14 breakpoints were introduced randomly into the sequence (seven paralog pairs where only one copy is associated with TE sequence) and an association was considered if the breakpoint was within 300 bp. This distance was also chosen to be conservative, given the distances observed in our data. 10,000 iterations were run and the upper 5% tail was calculated from the resulting distribution.

## Supporting Information

Figure S1 The Alignments of Gene Duplicate Copies and Their Flanked Repetitive Sequences (114 KB PDF)

Figure S2 The Simulation Results of the TE Association with Gene Duplications Vertical red line indicates the observed TE-associated genes in our paralog set. The distribution is from simulation where the mean TE-free regions are 15 kb, the mean distance at which our observation is significant at the 0.05 level [44].
(3 KB PDF)

Table S1 Expression Pattern of the New Genes and Their Parental Genes (135 KB DOC)

Table S2 Substitutions between Paralogous Copies The *p*-values in black are for the tests of the null hypothesis that Ka/Ks is significantly lower than 1. The *p*-values in red are for the null hypothesis that Ka/Ks is significantly lower than 0.5. *p*-Values for paralog comparisons (red) are shown only when the Ka/Ks value is lower than 0.5.
(56 KB DOC)

Table S3 Substitutions between Orthologous Copies The *p*-values are for the tests of the null hypothesis that Ka/Ks is significantly lower than 1.
(52 KB DOC)
