# Supplementary material for: Repetitive Element-Mediated Recombination as a Mechanism for New Gene Origination in Drosophila
Source: PLoS Genet. 2008 Jan 18;4(1):e3. doi: 10.1371/journal.pgen.0040003 (PMC2211543; doi:10.1371/journal.pgen.0040003)
Supplement: Supplementary file 1 [file pgen.0040003.sg001.pdf]

1. mkg-r/mkg-r2

```
                              5’breakpoint
mau-mkg-r         -TGGTAAATATCAAGTTTTCGGCA AAAGCCGTTTTTCCGAATTTCGGTCATTAAATAATC
mau-mkg-r2        TTAGAAAATTCCCAATTTT-GCCC AAAGCCGTTTTTCCCAATTTCTGCCATAAAATAATC
                  SAR_DM
mau-mkg-r         CGTTTTTTTGCCTCAACTTTAAAAATAATTGTCTGAATATGGAATGCCCTATCTCGTTGT
mau-mkg-r2        AGTTTTTTTACGAAAACTTTTAAAATAATTGTCTGTATATGGAAT----------GTTGA

mau-mkg-r         GCTCGTATTAAAATCCCCAATCGGAACTGTAGCCACAAATGGAAATTCTATTTTTTGGCC
mau-mkg-r2        GCTCGTAATTAAATTTCCAATC-AAACTGTGTTCAAAAATGCAAATTCTATTTTTTGGCC

mau-mkg-r         ATTTTTTGCAAATTTTGATGATGTTACCCCTTTCCAAAAATGCGAAAATTGACCCAAAAA
mau-mkg-r2        ATTTTTTGCAAATTTTGATGATGTTACCCCTTTCTAAAAATGCAAAAATTGACCCAAAAA

mau-mkg-r         TTAATTTTCCTAAATTCTTCAAAAAGTGATAGGGATTGTTAGCACTTGGTAAATATCTGC
mau-mkg-r2        TTAATTTTTCTAAATTCTTCAAAAAGTTATAGGGATTGTTAGCAC-TGGTAAATAGCTGC

mau-mkg-r         TCAAAACAGTTATTCTTTCTTCTTTATCAGCATTTTTAGCCAAGTTATGGTGGGAACCCC
mau-mkg-r2        TCAAAACAGTTATTCTTTCTTCTTTATCAGCATTTTTAGCCAAGTTATGGTGGGAACCCC

mau-mkg-r         TCCAAAATAAATACGCATTTTATAAATAATTAAGGCAATTAAGGTTTTAAAGTAAAAGCT
mau-mkg-r2        TCCAAAATAAATACGCATTTTATAAATAATTAAGGCAATTAAGGTTTTAAAGTAAAAGCT

mau-mkg-r         TCACAAATTTTTATTTATAATTATAGGGGCGGTAAATGAAGTAGTAATTGTTCAAAGGAT
mau-mkg-r2        TCACAAATTTTTATTTATAATTATAGGGGCGGTAAATGAAGTAGTAATTGTTCAAAGGAT

mau-mkg-r         AACGCTTTAATTCGATTTCGTACGCAGATTGTAAAGAAAGAGTAGTCGACTGAAATTCTA
mau-mkg-r2        AACGCTTTAATTCGATTTCGTACGCAGATTGTAAAGAAAGAGTAGTCGACTGAAATTCTA

mau-mkg-r         AAAAATTCACATCTAAGGAACATTTTGACCTGCCATATGTGAACTGTCAAAACAATACTT
mau-mkg-r2        AAAAATTCACATCTAAGGAACATTTTGACCTGCCATATGTGAACTGTCAAAACAATACTT

mau-mkg-r         CTTTATTGAGACGTTACAAAGTGAGCTAGTAAGAAATCGTAGCTGTCACAGAAAAAAACC
mau-mkg-r2        CTTTATTGAGACGTTACAAAGTGAGCTAGTAAGAAATCGTAGCTGTCACAGAAAAAAACC
                                                                     Start Codon
mau-mkg-r         TGTCTACATTTTTTGATTCTTCTTTTTGCGTAAATAAAAAA ATG TCGGCCTCTGCATCGG
mau-mkg-r2        TGTCTACATTTTTTGATTCTTCTTTTTGCGTAAATAAAAAA ATG TCGGCCTCTGCATCGG

mau-mkg-r         AATCAACTTCTCCGCCTGGGGATGGTGACGGTAATCTTGTGCTTTGCATGGTATGCGGTG
mau-mkg-r2        AATCAACTTCTCCGCCTGGGGATGGTGACGGTAATCTTGTGCTTTGCATGGTATGCGGTG

mau-mkg-r         CCCCCTTCCAGAGTACGCAGGATTGCCTTGCCCACGCGCTGCTCAAGCATGGCAGCAAGC
mau-mkg-r2        CCCCCTTCCAGAGTACGCAGGATTGCCTTGCCCACGCGCTGCTCAAGCATGGCAGAAAGC

mau-mkg-r         TCCAGAAGCAACTGCGAAAGCGATTGAACGCCATTACCACGATATTTAACAGCGCACAAA
mau-mkg-r2        TCCAGAAGCAACTGCGAAAGCGATTGAACGCCATTACCACGATATTAAACAGCGCACAAA

mau-mkg-r         GCCAATCCGAACGCCATGAGCTGCGGGAGGCTCTCGAGAAGTCCAAGCCCGGCGGGCACC
mau-mkg-r2        GCCAATCCGAACGCCATGGGCTGCGGGAGGCTCTCGAGAAGTCCAAGCCCGGCGGGCACC

mau-mkg-r         TGCGCACCGTATTGAATATCTTTGCTGTTGATCTCGAAAAGATGAAAACCTGCTTCGAGC
mau-mkg-r2        TGCGCACCGTATTGAATATCTTTGCTGTTGATCTCGAAAAGATGAAAACCTGCTTCGAGC

mau-mkg-r         ACGTCCGCAACTGCATAGAGAAGGAGATGAAGGGCAAGGTTAGGGTGTTCCCCTTTGGTT
mau-mkg-r2        ACGTCCGCAACTGCATAGAGAAGGAGATGAAGGGCAAGGTTAGGGTGTTCCCCTTTGGTT

mau-mkg-r         CACTGGTGACTGGTTTAGCATTGAAGGAGAGCGACCTGGATTTGTTTCTGGAGCCCAACG
mau-mkg-r2        CACTGGTGACTGGTTTAGCATTGAAGGAGAGCGACCTCGATTTGTTTATGGAGCCCAATG

mau-mkg-r         GCAACCAGCCACCATTGTGCCATCAGTATCTATACAACAGGACTTCATACTTTTTACGAA
mau-mkg-r2        GCAACCAGCCACCATTGTGCCATCAGTATCTATACAACAGGACTTCAAACTTTTTACGAA

mau-mkg-r         GTTCCAAATGCTTTGCAGATGTGGTAACCATTCGTCATGCCAGCGTGCCCATCATTAGAT
mau-mkg-r2        GTTCCAAATGCTTTGCAGATGTGGACACCATTCGTCATGCCAGCGTGCCCATCATTAGAT

mau-mkg-r         GTAAGCATCAGCTCACCGGACTGAACATAGACTTCAATATGTCAAATCCGAATGGCATAT
mau-mkg-r2        GTAAGCATCAGCTCACCGGACTGAACATAGACTTCAATATGTCAAATCCGAATGGCATAT
```

```
mau-mkg-r        TTAACTCCCGATTTGTGGGCGAACTGATGCTTCGCAATGAGCGGATACGCGAGCTCAGCT
mau-mkg-r2       TTAACTCCCGATTTGTGGGCGAACTGATGCTTCGCAATGAGCGGATACGCGAGCTCAGCT

mau-mkg-r        TATTTTTGAAAATCTGGGCCAAGAAGCTAAAATTGATTTGCAATGGCGGCATGACAAGCT
mau-mkg-r2       TATTTTTGAAAATCTGGGCCAAGAAGCTAAAATTGATTTGCAATGGCGGCATGACAAGCT

mau-mkg-r        ATTGCTTAATATCCTTGATCATCGTAAACTTGCAAGTGAATCGAATAGTTCCATCCGTCA
mau-mkg-r2       ATTGCTTAATATCCTTGATCATCGTAAACTTGCAAGTGAATCGAATAGTTCCATCCGTCA

mau-mkg-r        AACAGCTCCAGTCACTCTGTCCACCGGTTATTTTGTCGGGCGTTAACTTTGCCTACAGCC
mau-mkg-r2       AACAGCTCCAGTCACTCTGTCCACCGGTTATTTTGTCGGGCGTTAACTTTGCCTACAGCC

mau-mkg-r        TGGACCTAACGCCGCCGATTACAGAACGGCTCACCACGCTGGATTTACTTAAAAACTTCT
mau-mkg-r2       TGGACCTAACGCCGCCGATTACAGAACGGCTCACCACGCTGGATTTACTTAAAAACTTCT

mau-mkg-r        TCATATACTACAGCACTGTCAACTTTGACAAAAGTTTATTGAGTCCGTTTCTGGGCGGCT
mau-mkg-r2       TCATATACTACAGCACTGTCAACTTTGACAAAAGTTTATTGAGTCCGTTTCTGGGCGGCT

mau-mkg-r        GTGTGGACAAGGAGACAACACTGGGCATACCAGGAGGATTTCCCGAGTACGATGAGCAAC
mau-mkg-r2       GTGTGGACAAGGAGACAACACTGGGCATACCAGGAGGATTTCCCGAGTACGATGAGCAAC

mau-mkg-r        AGAAGCTCGTGCATGATGCAACAGGTGAGCCGCCACACGCTTTCCAGCTGGATAGAGTTA
mau-mkg-r2       AGAAGCTCGTGCATGATGCAACAGGTGAGCCGCCACACGCTTTCCAGCTGGATAGAGTTA

mau-mkg-r        TGTGCGTACAGGATCCCTTCGAGCTGAACCGCAACGTGGCCAAATCAGTGTCCATTGCAA
mau-mkg-r2       TGTGCGTACAGGATCCCTTCGAGCTGAACCGCAACGTGGCCAAATCAGTGTCCATTGC-A

mau-mkg-r        ATTTATTTTACTTTAGACAATGCCTAGTCCTGGCTGCTCAAGCATGCAGCGATCAGGAAC
mau-mkg-r2       ATTTATTTTACTTTAGACAATGCCTAGTCCTGGCTGCTCAAGCATGCAGCGATCAGGAAC

mau-mkg-r        TAACTTCGCAACCAGAGAAGCTATACGACTACCTATTATTTGGCCTGGCGGATAAGTTAG
mau-mkg-r2       TAACTTCGCAACCAGAGAAGCTATACGACTACCTATTATTTGGCCTGGCGGATAAGTTAG

mau-mkg-r        TAGCCGACAAGATAGTGGCAGATAAAAGATGGCAGATTCAATGCCAAATTGAAAACAGAT
mau-mkg-r2       TAGCCGACAAGATAGTGGCAGATAAAAGATGGCAGATTCAATGCCAAATTGAAAACAGAT
            Stop Codon
mau-mkg-r        CT TAA TGACTTACACTTTTCACACATTCAATTACTTGAATTTACAATATCACTATAGACT
mau-mkg-r2       CT TAA TGACTTACACTTTTCACACATTCAATTACTTGAATTTACAATATCACTATAGACT

mau-mkg-r        AAACTAATACACTAAACTAATAAACTAAACTAATAAAGTTGACTAAACTTTTTCTTGGCA
mau-mkg-r2       AAACTAATACACTAAACTAATAAACTAAACTAATAAAGTTGACTAAACTTTTTCTTGGCA

mau-mkg-r        AAAGTCTCTTGGAGCAGGGAGAAATTATTTACAAGGGCAACAAAGCTTACTTAAATCATT
mau-mkg-r2       AAAGTCTCTTGGAGCAGGGAGAAATTATTTACAAGGGCAACAAAGCTTACTTAAATCATT

mau-mkg-r        TACAAGGGTGTGTCTGAATAATAAAGAAGTTATAATTGATGGCCAGTTATCCAGCGCGTC
mau-mkg-r2       TACAAGGGTGTGTCTGAATAATAAAGAAGTTATAATTGATGGCCAGTTATCCAGCGCGTC

mau-mkg-r        TGAAGAAGGTCTGATCGTGACAGCGCCACTGTGGTTCAAAGCACTCATTAAGATCCTGCG
mau-mkg-r2       TGAAGAAGGTCTGATCGTGACAGCGCCACTGTGGTTCAAAGCACTCATTAAGATCCTGCG

mau-mkg-r        TCTATTCGTGCGAAAGAAGCTGCGCGAGCGAGTGTTCCCTGTATCGGTGCCCCAATTGGC
mau-mkg-r2       TCTATTCGTGCGAAAGAAGCTGCGCGAGCGAGTGTTCCCTGTATCGGTGCCCCAATTGGC
                                                                3'breakpoint
mau-mkg-r        GCTGCACGTGCCGCGCAAGACGCTGCCCCTTCAGTTGGGTGGCACGCTGGAGGTCGATCG
mau-mkg-r2       GCTGCACGTGCCGCGCAAGACGCTGCCCCTTCAGTTGGGTGGCACGCTGGAGGTCGATCG
```

2. CG3101/CG3101-r

```
sim-CG3101         ------------------------------------------------------------
sim-CG3101-r       AATGCAAATATGAAAATACCTTTCTGAGCTCGTAATTAAATTTCCAATCGAACTGTAATA

sim-CG3101         ------------------------------------------------------------
sim-CG3101-r       AGAAATAGGAATTCTATTTTTAATCAATTTTATACGTTTTGGCAAAGCGGTTTTTCTGAA
                   SAR_DM                                  SAR_DM
sim-CG3101         ----------------------------------------AACATAATTGCCTGAATATG
sim-CG3101-r       TTTCGGGCATAAAATAATTAGTATTTTGGGCACAGCACAAAAAATATTTGTTCAAATAAT
                   5'breakpoint
sim-CG3101         GAATGC CATACATCGTTGAGCTCGTAATTAAATTTCCAATCAAACTGTGTTCAAACATGG
sim-CG3101-r       GAAAGT CATAGCTCGTCGATATCGTTATTAAATTTCCAATCAAACTTTGTTCAAAAATGC

sim-CG3101         ATATTCAAATTTTTTGCCATTTT-------------------------------------
sim-CG3101-r       AAATTCTAATTTTTCGCCATTTTTTGCAAATTTTGATGATGTTACCCCTTACAAAAAAATG
```

```
sim-CG3101        ------------------------------------------------------------
sim-CG3101-r      CTAAAATTGATTCAAAAATTAATTTTCCTAAATTCTTCAAAAAGTGATAGGGATCGTTAG

sim-CG3101        -------------------------------------------------TTTTTAGCCAA
sim-CG3101-r      CACTGGTAATTAGCTGCTCAAAACAGTTATTCTTTCTTCTTTATGAGCATTTTTAGCCAA

sim-CG3101        GTTATGGTGGGAACCCTTCGAAAATAAATACGCATTTTATAAATAATTAAGGTAATTAAG
sim-CG3101-r      GTTATGGTGGGAACCCCTCGAAAATAAATACGCATTTTATAAATAATTAAGGTAATTAAG

sim-CG3101        GTTTTAAAGTAAAAGCTTCACAAATTTTTATTTATAATTATAGGGGCGGTAAATGAAGTA
sim-CG3101-r      GTTTTAAAGTAAAAGCTTCACAAATTTTTATTTATAATTATAGGGGCGGTAAATGAAGTA

sim-CG3101        GTAATTGTTCAAAGGATAACGCTTTAATTCGATTGCGTATGCAGATTGTAAAGTAGTCGA
sim-CG3101-r      GTAATTGTTCAAAGGATAACGCTTTAATTCGATTTCGTATGCAGATTGTAAAGTAGTCGA

sim-CG3101        CTGAAATTCTAAAAAATTCAGATCTAAGGAACATTTTGACCTGCCATATGAGAACTGTCA
sim-CG3101-r      CTGAAAATCTAAAAAATGCAGATCTAAGGAACATTTTGAC--------------------

sim-CG3101        AAACAATACTTCTTTATTGAGACGTTACAAAGTGAGCTAGTAAGAAATCGTAGCTGTCAC
sim-CG3101-r      -----------CTTTATTGAGACGTTACAAAGTGAGCTA-TAAGAAATCGTAGCTGTCAC
                                                         Exon4
sim-CG3101        AGAAAAAAACCTGTCTAAATTTTTTGATTCTTGATG GCCGGTTATACAGCGCGTCTGAAG
sim-CG3101-r      AAAAAAAAACCTGTCTAAATTTTTTGATTCTTGATG GCCGGTTATACAGCGCGTCTGAAG

sim-CG3101        AAGGTCCTGATCGTGACAGCGCCACTGTGGTTCAAAGCACTCATTAAGATCCTGCGTCTA
sim-CG3101-r      AAGGTCCTGATCGTGACAGCGCCACTGTGGTTCAAAGCACTCATTAAGATCCTGCGTCTA

sim-CG3101        TTCGTGCGAAAGAAGCTGCGCGAGCGAGTGTTCACTGTATCGGTGCCTCAATTGGCGCTG
sim-CG3101-r      TTCGTGCGAAAGAAGCTGCGCGAGCGAGTGTTCACTGTATCGGTGCCTCAATTGGCGCTG

sim-CG3101        CCCCTTCACTTGGGTGGCACGCTGGAGGTCGATCACGACACATCGTTGCTCAGCTGACGC
sim-CG3101-r      CCCCTTCACTTGGGTGGCACGCTGGAGGTCGATCACGACACATCGTTGCTCAGCTGCCGC

sim-CG3101        CAATCGATGATGAATCGTGAGGACAAGCTGCTGGCCAATATCATGGATGTGAGCGATGGA
sim-CG3101-r      CAATCGATGATGAATCGTGAGGACAAGCTGCTGGCCAATATCATGGATGTGAGCGATGGA

sim-CG3101        TCCGGATCCAGTTCAGAAACTGGATCAGAAATTGTGTCCAACAGTCAAAAAGCCAGGATA
sim-CG3101-r      TCCGGATCCAGTTCAGAAACTGGATCAGAAATTGTGTCCAACAATAAAAAAACCAGGATA

sim-CG3101        ATCAGCGCCGTCCACCAGTTGGGCGATAATAATACCGCGGACAACAACAGAACCATCACG
sim-CG3101-r      ATCAGCGCCGTCCACCAGTTGGGCGATAATAATACCGCGGACAACAACAGAACCATCACG

sim-CG3101        TACGCCACCACAGCCTCTGGAGCAGCTAACGGCGCCACCACCAATGGGGGCAGACAGTTT
sim-CG3101-r      TACGCCACCACAGCCTCTGGAGCAGCTAACGGCGCCACCACCAATGGGGGCAGACAGTTT

sim-CG3101        TTGAACCCGCCCAGCAGCTCCTCATCCGACATGAGTATCGATGATAGTCTCGATGGTCAG
sim-CG3101-r      TTGAACCCGCCCAGCAGCTCCTCATCCGACATGAGTATCGATGATAGTACTGATGGTCAG

sim-CG3101        GAGGGCGATTCCAAGTCCATTCATCAGATTGTCCAGATGATAAAGCAGGGCGGACTCCGT
sim-CG3101-r      GAGGGCGATTCCAAGTCCATTCATCAGATTGTCCAGATGGTGAAGCAGGGCGGACTCCGT

sim-CG3101        GGTCTGTTTGAGGAGTACGCGGTCATTCGGAATAGGCCGCCCGAGGGCACCTTCTGGCAT
sim-CG3101-r      GGTCTGTTTGAGGAGTACGCGGTCATTCGGAATAGGCCGCCCGAGGGCACCTTCTGGCAT
               Exon4
sim-CG3101        TCAAG GTAGGTCAAATGAAATCTTCATTCTAGATCACTGATAATTAACGTTCCTATATTC
sim-CG3101-r      TCGAG GTAGGTCAAATGAAATCTTCATTCTAGACCACTGATAATTAACGTTCCTATATTC
                        Exon5
sim-CG3101        TCGCAG AATGCATGCCAATCTGACCAAGAATCGCTTCGCAGACGTCCTCTGCTACGATCA
sim-CG3101-r      TCGCAG AATGCATGCCAATCTGACCAAGAATCGCTTCGCAGACGTCCTCTGCTACGATCA

sim-CG3101        AAATCGAGTGGTTCTGACACACGAAGACGGCGACGAGGCGTCGGATTACATAAATGCGAA
sim-CG3101-r      AAATCGAGTGGTTCTGACACACGAAGACGGCGACGAGGCGTCGGATTACATAAATGCGAA
                                                            Exon5
sim-CG3101        CTTCGTCGATGGCTACAAAGAAAATAGTGCCTATATAACAACTCAAG GTAAGCGATGATG
sim-CG3101-r      CTTCGTCGATGGCTACAAAGAAAATAGTGCCTATATAACAACTCAAG GTAAGCGATGGTG
                                                                       Exon6
sim-CG3101        ACACACTAAATCATATGCATAAACTATATGCTAATGGAGTTAACTCCGCCAG GTCCATTA
sim-CG3101-r      ACACACTTAATCATATGCATAAACTATATGCTAATGGAGTTAACTCCGCCAG GTCCATTA

sim-CG3101        CCAAACACATGCAAGGACTTTTGGCGCATGATCTGGGAACAACATTGTTTACTTATAGTG
sim-CG3101-r      CCAAACACATGCAAGGACTTTTGGCGCATGATCTGGGAACAACATTGTTTACTTATAGTG
```

```
sim-CG3101       ATGACGATGCGCGTGGTGGAGCGCGGATGTGTGAAATGCGTCCAGTACTGGGAGCCGACT
sim-CG3101-r     ATGACGATGCGCGTGGTGGAGCGCGGATGTGTGAAATGCGTCCAGTACTGGGAGCCGACT

sim-CG3101       GAAGAAGGTTCCTTAGAGTTTGGCGACTACCATGTGCGAACAATTAGCGTTGAGTGCAAC
sim-CG3101-r     GAAGAAGGTTCCTTAGAGTTTGGCGACTACCATGTGCGAACAATTAGCGTTGAGTGCAAC

sim-CG3101       GAGGGCTACATGGTTACCTCGTTAGAATTGAGAAACATAAAG------------------
sim-CG3101-r     GAGGGCTACATGGTTACCTCGTTAGAATTGAGAAACATANNNNNNNNNNNNNNNNNNNNN
                                           Exon6
sim-CG3101       -------------------------- GTGAAGCTCTCTTTATAGTACAACGATAGCGAATA
sim-CG3101-r     NNNNNNNNNNNNNNNNNNNNNNNNNN NNNNNNNNNNNNNNNNNNNNNNNNNNNNNNNNNNNN
                                                            Exon7
sim-CG3101       TTATTAGAATCACTTTGTCATTTCGCCAACAG ACAGACGAAGTTCGCAATGTCTCACACT
sim-CG3101-r     NNNNNNNNNNNNNNNNNNNNNNNNNNNNNNNN NNNNNNNNNNNNNNNNNNNNNNNNNNNNN

sim-CG3101       GGCAGTTCACCAGCTGGCCGGACTATGGCATTCCCAGCTCGGCCATGACCATGCTGAACT
sim-CG3101-r     NNNNNNNNNNNNNNNNNNNNNNNNNNNNNNNNNNNNNNNNNNNNNNNNNNNNNNNNNNNN

sim-CG3101       TCGTGCAGAAGGTGCGTGAGAAGCAGGCGCAGGTCCTCGAAGGACTGGTCGACACCTGGG
sim-CG3101-r     NNNNNNNNNNNNNNNNNNNNNNNNNNNNNNNNNNNNNNNNNNNNNNNNNNNNNNNNNNNN
                                                                              Exon7
sim-CG3101       CGGGTCATCCCCTTGGACCGCCCATTGTGGTGCACTGCAGTGCAGGCATTGGACGCTCAG
sim-CG3101-r     NNNNNNNNNNNNNNNNNNNNNNNNNNNNNNNNNNNNNNNNNNNNNNNNNNNNNNNNNNNN

sim-CG3101       GTGAGTCGCCAACATTGTAGACAATCCACGCCAGATTGACCGACTACCAAAAACTCTACA
sim-CG3101-r     NNNNNNNNNNNNNNNNNNNNNNNNNNNNNNNNNNNNNNNNNNNNNNNNNNNNNNNNNNNN
                       Exon8
sim-CG3101       CAG GTACATTCATCACCCTGGCCACTTGCATATCGCGCCTGGAGGACGTGGGCACGGTGG
sim-CG3101-r     NNN NNNNNNNNNNNNNNNNNNNNNNNNNNNNNNNNNNNNNNNNNNNNNNNNNNNNNNNNN

sim-CG3101       ACATACGTGGAACCGTTGAAAAGATCCGCTCGCAGCGAGCCCACTGTTTTCAGATGCCCG
sim-CG3101-r     NNNNNNNNNNNNNNNNNNNNNNNNNNNNNNNNNNNNNNNNNNNNNNNNNNNNNNNNNNNN

sim-CG3101       AACAGTACGTGTTCTGCCACCTGGCCCTCATCGAATACGCATATTCGCGTGGAATGCTAA
sim-CG3101-r     NNNNNNNNNNNNNNNNNNNNNNNNNNNNNNNNNNNNNNNNNNNNNNNNNNNNNNNNNNNN
                                                                  Stop Codon
sim-CG3101       AGCCGGCCGATCTGGCTGGCTTCTATCAGCATGAACCGGACTTGGAG TAG GCTAATACTA
sim-CG3101-r     NNNNNNNNNNNNNNNNNNNNNNNNNNNNNNNNNNNNNNNNNNNNNNN NNN NNNNNNNNTA

sim-CG3101       CCAACACAGTCCTTGGACCGGAAAACGGACGAGGAACCAACTTGAATACAGAATAATTTT
sim-CG3101-r     CCAACACAGTCTTTGGCCGGGAAACGGGAGAGGGAACCAACTTGTTTACAGAATAATTTT

sim-CG3101       ATGCTTTACCATTGCAAATTGGATTTGCAACTTTCTTCGACTA-GATGTTGGTCTTGATG
sim-CG3101-r     ATGTTTTACCATTGCAAATTGGAGTGGCAACTTTCTTCGACTAGGATGTTGGTCTGGATG

sim-CG3101       TGATA-TAATATGATATATATGATACGAGAGAAACGATGGGGAGCACAAATAGATCCAGA
sim-CG3101-r     TGATATTAATATGATATATATGATACGAGAGAAACGATGGGGAGCACAAATGGATCCAGA

sim-CG3101       GACACGGAGAGCGAGATGAAGTTGTGGGGAGTAGAGTAACCAATTTACGTTAATCTTTTA
sim-CG3101-r     GACACGGAGAGGGAGATGAAGTTGTGGGGAGTAGAGTAACCAATTTACGTTAATCTTTTA

sim-CG3101       TTTTTTTAAATATTAATACATACCAAGCAAAAATTGGTA-----------TACATAAATT
sim-CG3101-r     TTTTTTAAAATATTAATACATACCAAGCAAAAATTTCTATGTTAAAACGTTACATTAATT

sim-CG3101       AAATTTAAAGTATGTGAAAAACAAATAGCAAAAAATTTAGAAACAAAACCAGCAACATTA
sim-CG3101-r     AAATTTAAAGTAAGTGAAAAACAAATAGCAAAAAATTAAGAAACAAAACCAGCAACATTA
                                                         Polyadenylation Site
sim-CG3101       GCAAAACAAACAGC----AAAGAGTTCTTAACTGAAGAATAATTTCTTGGATTT CTGGTA
sim-CG3101-r     GCAAAACAAACAGCAAAGAAAGAGTTTTTAACCGAAGAATAATTTCTTGGATTT CTGGTA

sim-CG3101       CCAGGCGATCATAATCATTAGCATGTCGGGCATAACTTAAAAAATAATCGCCTGAATATG
sim-CG3101-r     CCAGGCGATCATAATCATTAGCATGTCGGGCATAACTTAAAAAATAATCGCCTGAATATG
                                             SAR_DM
sim-CG3101       GAATGTTATGCCTCGTTGAGCTCGTAATTAAATTTCCAATCAAACTGTTTTCAAATATGA
sim-CG3101-r     GAATGTTATGCCTCGTTGAGCTCGTAATTAAATTTCCAATCCAACTGTGTTCCAAAAGGT

sim-CG3101       AAATTCTATTTTTTGTCAATTTTTTGCAAATTTTGATGATGTTACCCCTTTCCAAAAATG
sim-CG3101-r     TAATTCTATCTTTTGGCCATTATTTGCAAATTTTGATGA---------------------

sim-CG3101       CGAAAATTGACCCAAAAATTGATTTTCCTAAATTCTTCAAAAAGTGATAGGGATCGTTAG
sim-CG3101-r     -AAAAATTCACCCAAAAATTAATTTTCCTAAATTCTTCAAAAAGTGATAGGGATCGTTAG
```

```
sim-CG3101        CATTGGTAATTAGCTGCTCAAAACAGTTATTCTTACATCTATATGACAATTTTCAGCCAA
sim-CG3101-r      CATTGGTAATTAGCTGCTCAAAACAGTTATTCTTTCATCTTTATGACAATTTTCAGCGAA

sim-CG3101        GATATGACGAAAATTCCGTTTGTAATTAACACGTTTTTGGCAAAAGCGGTTTTTCCGAAT
sim-CG3101-r      GGTATGACCATAATTCCATTTGTAAATATCAAGTTTTTGGCAAAAGCTGTTTTTCCGAAT

sim-CG3101        TTCGGTAGTCAAATAATCAGTTTTTCCGACACAACTTGAAATTTAATTGTCCAAATCTGG
sim-CG3101-r      TTCGGTAGTCAACTAATCAGATTTTTCGACACAACTTTAAAAATAATTGTCCAAATATGA

sim-CG3101        AATGCTATACCTCGCTGAACTTGTAATTAAATTTCCAATCAAACTGTGTTCAAAAATGCA
sim-CG3101-r      AATGCCATATCTCGTTGTGTTCGTAATTAAATTTCCAATCGAACTGTATCCACAAATGGA

sim-CG3101        AATTCTATTTTTTCCCCATTTTTTGCAAATTTTGATGATGTTACCCCTTACAAAAAATGC
sim-CG3101-r      AATTCTATTTTTTGGCAATTTTTTGCAAATTTTGATGATGTTACCCCTTACAAAAAATGC

sim-CG3101        AAAAATTCACCCAAAAATTAATTTTCCTAAATTCTTCAAAAAGTGATAGGGATTGTTAGC
sim-CG3101-r      AAAAATTCACCCAAAAATTAATTTTCCTAAATTCTTCAAAAAGTGATAGGGATCGTTAGC
                                                                3'breakpoint
sim-CG3101        ACTGGTAATTAGCTGCTCAAAACAGTTATTCTTTCTTCATTATGAGCATTT▼ TAGCCAGG
sim-CG3101-r      ATTGGTAATTAGCTGCTCAAAACAGTTATTCTTTCATCTTTATGAGCATTTT --------

sim-CG3101        TTATGACTAAAATTCCATTCGAAAAGTTTTGGATTTTTCGAAAATCTGGATCCAAAATCC
sim-CG3101-r      ------------------------------------------------------------

sim-CG3101        GTAAATCTGGATTGCAAACCTGTCCAAATCTGGATTGCATTCCTCGTTGAGCTTGTTATT
sim-CG3101-r      ------------------------------------------------------------

sim-CG3101        AAA
sim-CG3101-r      ---
```

3. kep1/CG3875

```
mel-kep1        ------------------------------------------------------------
mel-CG3875      GGAAGCGTTTCCGACCATATTAAGTATATTCTTGATATATTATATTATGAATATTCTTAA
                DNAREP1_DM
mel-kep1        ------------------------------------------------------------
mel-CG3875      TTAGATATATTCTTGATCAGAATCAATATCAACTGTCTGTCCTTCTGTAGGAACGTCGAG

mel-kep1        ------------------------------------------------------------
mel-CG3875      ATCCCAGAAACTATAAAAGCTACAATGTTGAGATAAGGGTCACAGCTTCCCGAGACATAG

mel-kep1        ------------------------------------------------------------
mel-CG3875      TCGCAGCCTTTGTTGAACCATGTACCACGCCCACTCTAACGCCCACAAACATAATGGGCA
            5'breakpoint
mel-kep1        --▼CGACATTTAAACAATCACTATTGAATTAAAAAAGTTGGCAGCCCGAAAACTTCACACG
mel-CG3875      AC CGACATTTAAACAATCACTATAGCACTGAAAAAGCTGACAACCT-CAAACTTCATACG
                         Transcription Start Position
mel-kep1        CTTGGC AGCTCTGCCATTATTTTAGTTCATTGGTGTACATACTGTTTTTCAGTCTACACC
mel-CG3875      TTTGGC AGCTCGGCCAGCATTTCAGTTCATTGATATACAGAACATTGTTCAGTCTACATC
                                                         Start Codon
mel-kep1        -ATTTTTAATTGT-AATTTTCTGTAAAGATTGTT ATG ATAAAAATGGAAACCCCAAGCGA
mel-CG3875      AATTTTGAATTGTGAATATTCGGTAAAGATTATT GTG ATAAAAATGGAAACCCAAAGCGA

mel-kep1        GTTTACTGAGAAACAG---------CCACCCACCCACGATC---------ACCAGCCGCG
mel-CG3875      GTTTACTGAGGAACAGAACCAAGACCAACCCACCCAGGACCAACCTACCTACCAGCCGCG

mel-kep1        TCTTAACGAGGTGGCCCAAAAGTTTCTCGCCGATTTGGACGAGGAGCGCCAGCGATTGTC
mel-CG3875      TCTGAACGAGGTGGCCCAAAAGTTTCTCGCCGATTTGGACGAGGAGCGCAAGCGATTGTC
                                                    Exon1
mel-kep1        CGCGGACTTTCCACTTTGCGCACTGCTAATCGACGAGG GTAAAATCAATTTGGCCCTTTT
mel-CG3875      CGCGGAGTTTCCACTTTGCGCTCTGCTAATCGACGAAT GTAAAATCAATTTGGCTC-TTT
                                                         Exon2
mel-kep1        ACAGGGTATTTTAATGGTACTAATCCCAATATTGCAG CTGTGGACCGTGTCTACTGCACT
mel-CG3875      ACAGGGTATTTTAATGGTACTTATCCCAATATTGCAG CTGTGGACCGTGTCTTCTCCACT

mel-kep1        GGTCGTATTCCCGGAAAAGAGTTCTACGCAGACGTGTACAAGCAGAAGCCGATGAAGATT
mel-CG3875      GGTCGTATTCCCGGCAAAGAGTTCTACGCAGACGTGTACCACCAGAGGCCGATGAAGATT
                                                     Exon2
mel-kep1        ACCCAAAAGGTCTTCGTGCCCGTAAAGCAGTATCCCAAG GTGGG-TTTATACAATTAGCT
mel-CG3875      ACCCAAAAGGTCTTCGTGCCCGTTAATAAGTTTCCCAAG GTGGGTTTTACACAATTAGTT
                                                          Exon3
mel-kep1        GTCCTCTCCGTTCGAAAAATAAATCCGTACCCTTGCGCAG TTCAACTTCACTGGCAAGAT
```

```
mel-CG3875      GTCCCCTCCGTCCGCAAAATAAATCCGTACCCTTGCGCAG TTCAACTTCGCTCGGAAGAT

mel-kep1        CCTGGGGCCCAAGGGCAATTCGCTGCGTCGCCTTCAGGAGGAGACCCAGTGCAAGATTGC
mel-CG3875      CCTGGGGCCCAAGGGGAACTCGGTGCGTCGCCTAAAGGAGGAGACCAACTGCAAGATTGT

mel-kep1        CATTAAGGGTCGCAGTTCGATCCGCGATCGTAACAAAGAAGAGCAGCTGCGCAGCACAGG
mel-CG3875      CATCAAGGGTCGCAGCTCGATGCGCGATCGTAACAAAGAGGAGGAGCTGCGCAGCTCAGG

mel-kep1        CGACCCGAGATATGCTCATCTTCAGAAGGACCTATTTTTGGAGGTCAGCACGGTGGCCAC
mel-CG3875      CGACCCGAGATATGCCCATCTTCACAAGGATCTCTTTTTGGAGGTCAGCGCGGTGGCCCC

mel-kep1        TCCGGCGGAGTGCTATGCCCGCATAGCCTACGCTCTGGCCGAGATCCGTAAGTATCTTAT
mel-CG3875      TCCGGCGGAGTGCTATGCACGGATAGCCTACGCCCTGGCCGAGATCCGTAAGTACCTCAT

mel-kep1        TCCAGACAAGAACGACGAGGTTTCACACGAACAGCTGCGCGAGCTGATGGAAATGGATCC
mel-CG3875      TCCAGACGACAACGACGATGTTTGGCACGAGCAGCAGCGCGAGCTGATGGAAATGAATCC
                                                          Exon3
mel-kep1        CGAGTCAGCCAAAAACATTCACGGACCGAATCTGGAGGCCTACAG GTA------ATCTTA
mel-CG3875      CGAGTCGGCCAAGAAAAGTAACGGACTCAATATGGCGCCCTACAG GTAATGGTCATCTTA

mel-kep1        CACCCTATGCA-------------------------------------------------
mel-CG3875      TTCTCTCTGCATACCGATGCAACTTGACTTACTTGACAAGCAAGTTAAGCATGTGGTATC
                                                                       Exon4
mel-kep1        ---------GCCTTTGGGCATGAGAATAAAAATA---TATTATTTTGCAG ATCTGTCTTC
mel-CG3875      CATCTGCTTGCCATTGGGCA-AAGGGTAAAAATCCATTGTTATTTTGCAG ATCGATCTTC

mel-kep1        GACAAGAAGTTTGGAGGCAACAGCAATGGGGCTCCCAAATACATCAACCTGATTAAGAGA
mel-CG3875      GACAAGACGATTGGAGGCAACAGGAATGGGGCTCCCAAGTACAACAACCAGATTAGGCGA
                            Exon4
mel-kep1        GCTGCGGAAAATCCGCCCGA GTAAGTTACTTGTAAAGTATAATTTTGTATTATTTAATGA
mel-CG3875      GTTACGGAAAATCCGCGCGA GTAAGTTACTTGTAAAGTATAATTTTGTATCATTTAATGA

mel-kep1        CCCCACCAGGAGTAATGGAACCGAGAGTCCTCGCGTCATAGTAGTGCCTTAAATCCTAGC
mel-CG3875      CCAAACCAGGAGTTA---------------CCGCGTCCTAGTAGTTCCTTAAATCCTAGC
                                                                  Exon5
mel-kep1        CAACATCTGTTAACCCAGCAATTAACATTTTCATTTTTATAG AGTCGACGATGTGGAGGA
mel-CG3875      CAACATGCGTTGAGCCAGCAACTAACATTTTCATCTTTATAG AGTCGCCGATATGGAGGA

mel-kep1        GGTGGCCTATGAGT---ATGAACATCGTATGCCCCCCAAGCGTCCGCCTACGGGCTATGA
mel-CG3875      GGTGGAATATGATTATGATGAACATCGTATGCCCCCCAGTCGTCCGTCTTTGGGCTTTGA
                    Exon5
mel-kep1        GTACAGCAAAC GTAAGTTAATCCCAAGCATGCATTCTTTATTTGGGTAGTGCCGGGGTCT
mel-CG3875      GTATAGCAAAC GTAAGT-------------------------------------------

mel-kep1        ATGACATAAGATCGATTTTTCCCCTGAATGCACCAGTTTTTTGGGATGCACCTTTGACAG
mel-CG3875      ------------------------------------------------------------

mel-kep1        GAACTTTGGGTTATAGATTAGATTAGATCATACTATCAAAACCAATAACTGTGATTGAGC
mel-CG3875      ---------GTT---------ATTAGATTATAATATCAAAATCAATAATCG---------

mel-kep1        AAGCTCTCAGATCAAACTATATAATACACTGGAATGATTAATATTATGCAACGATTCACT
mel-CG3875      -------------AAGCTATGTAATATACTAGAATGAATAATATTATGAAACTAATAAAT

mel-kep1        CCTTTATAGTAATTACATACACGCATAAAATACTACAAATAATAATCAACCAATTTTATA
mel-CG3875      AAATAATAAATAATAATAATAATAATAAATCAATACAAATAATAATCAACCAATTTTATA
                    Exon6
mel-kep1        G CACGTCCATCAATAATACCGACAAACGCAGCGGCATATAAACG------TCCATATCCG
mel-CG3875      G CACCTCCATCAATGACAGCGACAAACGCTACGCCATTTAAACGTGCATATCCGTATCCG

mel-kep1        ACTGACATGAAACGTATGCGCGAACCGCCCATCAAGTCCTATAAGCCCAATCCGTATACA
mel-CG3875      ACTGACATGAATCGTACGCGCGAACCGCCCATCAAGTCATATAAGCCCAATCCGTATACA
                            Stop Codon
mel-kep1        ATACTTAAAAA—ATATAAA TAA TAGAGCTCCGGTTGATTTCGTCGCACACCGCTATAAA
mel-CG3875      ATACTTAACAAATATATAAA TAA TAGAGCGCCAAGTGATTTCGTCGCACAACGCTATAAT

mel-kep1        TGAAAACAAAGGACAATGCTTACGTTATAAAATCCATGCT-GGGAGCACATTGAAAATAC
mel-CG3875      TGAAAACAAAGGACAATGCTTACGTTGTAAAATCCATGCCGGGGAACTCATGGAA-----

mel-kep1        TTTAATCAAGAAGAAAGCAGCATTCAAAATGATGCTATCAATTAATAAACATTAATATTT
mel-CG3875      -------AAGAAGAAAGCAGCATTCAAAATGATGCTATCAATGAATAAA---------TT

mel-kep1        ATAAAACGCGCACTTGCGCTATCAATTCCCACAAACAATATTAATCTTAAATTTGTATAT
```

```
mel-CG3875      TTAAAAAAAGCACTTGCGCTGACAA-TCCTACAAGCAATATTAATTTGAATTTTTTAT--

mel-kep1        CCATAAATATGTTAAACTGTTGGCATGCCTTTTCTGGACCATTTCAACAGATAATAAGTC
mel-CG3875      ----------------------------------TGG-----------------------

mel-kep1        AAATAATTCCTATTATGTTTAAATTATGATTTCCCGAGTTCGTAATTTTCTATGAATG--
mel-CG3875      --------------------------------CCTGGATTCGTAATTTTCTATGAATGTA
                                                  Polyadenylation Site
mel-kep1        ------TAATAAAATGATACGCATTGAATGAATTTAATAAATTCTCTGCAATCAGA TCTA
mel-CG3875      AAAAACTAATAAAATAAAATGCATTGCATGAATTTGAAAAATTCTTTGAAATTAGA TCTA
               3'breakpoint
mel-kep1        ACTAT
mel-CG3875      ACTAT
```

4. CG3875/CG3927

```
sim-CG3875      -----GTAGAAG-AAAGCGTTTCCGACCATATTACGTATTTTCTTGATATATAATATTTA
sim-CG3927      GAGACATAGACGCAGCGCTTGTTTAACCACGCC---------------------------
                DNAREP1_DM
sim-CG3875      TATTAAGTATATTATAATATTAAAAATGTTCTTAAGTAAGTATAATTTTGATAGAATGTT
sim-CG3927      --------------------------CACTCTAAAGCC--CACAAATATAATGGAAAACC
                       5'breakpoint
sim-CG3875      GGGACTAGGAGCA CAATCACTATAGCACTGAAAAACTTGACAGCCTCAAACTTCATACGT
sim-CG3927      CGACATTTGAG-- CAATCACTATAGCACTGAAAAAGTTGACAGCCTCAAACTTCATAAGT

sim-CG3875      TTGGCAGCTCCGGCACCATTTCAGTTCAGTGTTATTCAGAACGTTGTTCAGTCTACATCT
sim-CG3927      TTGGCAGCTCCGAAACCATTTCAGTTCAGTGTTATTCAGAACGTTGTTCAGTCTACATCA
                                                           Start Codon
sim-CG3875      ATTTTAATTGTGATTTTTTCGGTAAAGTTCGTTGTGATAAAA ATG GAAAACCCAAGCGAG
sim-CG3927      ATTTTAATTGTGATTTTTTCGGTAAAGTTCGTTGTGATAAAA ATG GAAAACCCAAGCGAG

sim-CG3875      TTTACTGAG---------------------------------------------------
sim-CG3927      TTTACTGAGAGTTGTCCTGAGTTTACCCAGGAGCAACCTACCCGCCTGCCGCGTGTGAAC

sim-CG3875      ------------------------------------------------------------
sim-CG3927      GAGGTGGCCCAAAAGTTTCTCGCCGATTTGGACGAGGAACAGCCACACAACCAAGACCAA

sim-CG3875      ---------------------AAACAGCAACCTACCAAGGAGCAACCTACCCACCTGCCG
sim-CG3927      CCTACCCAGGAGCAACCTAGCAAGGAGCAACCTACCAAGGATCAACCTACCCACCTGCCG

sim-CG3875      CGTGTGAACGAGGTGGCCCAAAAGTTTCTCGCCGATTTGGACGAAGAGCGCAAGCGATTG
sim-CG3927      CGTGTGAACGAGGTGGCCCAAAAGTTTCTCGCCGATTTGGACGAGGAGCGCAAGCGATTG
                                                 Exon1
sim-CG3875      TCCGCGGAGTTTCCACTTTGCGCACTGCTAATCGACGAGG GTAAAATCAATTGCGCCCTT
sim-CG3927      TCCGCGGAGTTTCCACTTTGCGCACTGCTAATCGACGAGG GTAAAATCAATTGCGCCCTT
                                                        Exon2
sim-CG3875      TACAGGGTATTTTAATAGTACTAATCCCACATTGCAG CTGTGGACCGTGTCTACGGCACT
sim-CG3927      TACAGGGTATTTTAATGGTACTAATCCCACATTGCAG CTGTGGACCGTGTCTACAGCACT

sim-CG3875      GGTCATATTCCCGGCAAGGAGCGCTTCGCAGACGTGTACCAGCAGAAGCCGATGAAGATT
sim-CG3927      GGTCATATTCCCGGCAAGGAGCGCTTCGCCGACGTGTACCAGCAGAAGCCGATGAAGATT
                                                Exon2
sim-CG3875      ACCCAAAAGGTCTTCGTGCCCGTTAATCAGTTTCCCAAG GTGGGTTTTATACAATTAGCT
sim-CG3927      ACCCAAAAGGTCTTCGTGCCCGTTAATCAGTTTCCCAAG GTGGGTTTTATACAATTAGCT
                                                           Exon3
sim-CG3875      GTCCCCTCCGTCCGCAAAATAAATCCGTACCCTTGCGCAG TTCAACTTCGCTGGCAAGAT
sim-CG3927      GTCCCCTCCGTCCGCAAAATAAATCCGTACCCTTGCGCAG TTCAACTTCGCTGGCAAGAT

sim-CG3875      CCTCGGGCCCAAGGGGAACTCACTGCGTCGCCTTCATGAGGAGAGCCAGTGCAAGATTGC
sim-CG3927      CCTCGGGCCCAAGGGGAACTCACTGCGTCGCCTTCAGGAGGAGAGCAAGTGCAAGATTGC

sim-CG3875      CATTAAGGGTCGCAGTTCGATCCGCGATCGTAACAAAGAGGAGCAGCTGCGCAGCTCCGG
sim-CG3927      CATTAAGGGTCGCAGTTCGATCCGCGATCGTAACAAAGAGGAGCAGCTGCGCAGCTCCGG

sim-CG3875      CGACCGGAGATATGCCAATCTTGAGAAGAATCTCTTTCTGGAGGTCAGCACGGTGGCCC-
sim-CG3927      CGACCGGAGATATGCCCATCTTGAGTAAAATCTC-TTCTGGAGGTCAGCACGGTGGCCCN

sim-CG3875      ------------------------------------------------------------
sim-CG3927      NNNNNNNNNNNNNNNNNNNNNNNNNNNNNNNNNNNNNNNNNNNNNNNNNNNNNNNNNNNN

sim-CG3875      ------------------------------------------------------------
sim-CG3927      NNNNNNNNNNNNNNNNNNNNNNNNNNNNNNNNNNNNNNNNNNNNNNNNNNNNNNNNNNNN
```

```
sim-CG3875      ------------------------------------------------------------
sim-CG3927      NNNNNNNNNNNNNNNNNNNNNNNNNNNNNNNNNNNNNNNNNNNNNNNNNNNNNNNNNNNN

sim-CG3875      ------------------------------------------------------------
sim-CG3927      NNNNNNNNNNNNNNNNNNNNNNNNNNNNNNNNNNNNNNNNNNNNNNNNNNNNNNNNNNNN

sim-CG3875      ------------------------------------------------------------
sim-CG3927      NNNNNNNNNNNNNNNNNNNNNNNNNNNNNNNNNNNNNNNNNNNNNNNNNNNNNNNNNNNN

sim-CG3875      ---------------------------------CTCCGGCGGAGTGCTATGGCCGCATAG
sim-CG3927      NNNNNNNNNNNNNNNNNNNNNNNNNNNNNNNNNNNNNNNNNNNNNNNNNNNNNNNNNNNN

sim-CG3875      CCTACGCCCTGGCCGAGATTCGTAAGTATCTTATTCCAGACAAGAACGACGAGGTTTCAC
sim-CG3927      NNNNNNNNNNNNNNNNNNNNNNNNNNNNNNNNNNNNNNNNNNNNNNNNNNNNNNNNNNNC

sim-CG3875      ACGAGCAGCTGCGTGAGCTGATGGAAATGGATCCCGAGTTGGCCAAAAACACTAAAGGAC
sim-CG3927      ACGAGCAGTACCGCGAGTTGATGGAAATGAATCCCGAGATGGCCAAGAACTCTAACGCAC
                            Exon3
sim-CG3875      TCAATCTGCCGGCCTACAG GTAATGGGGATCTTATACTCTCTGCATACCGATGCA-----
sim-CG3927      TCAATCAGCCGGCCTACAG GTAATGGGGATCTTATACTCTCTGCATACCGATGCAGCTTC
                                                             Exon4
sim-CG3875      ----ACTTGACAAGCAAGTAAAGCATGTGGTATACAGCAG ATTGCCATTGGGCCAAGGGT
sim-CG3927      ACTTACTTGACAAGCAAGTAAAGCATGTGGTATACAGCTG ATTGCCATTGGGCCAAGGGT

sim-CG3875      AAAAATTTATAAATCTATTATTTTGCAGATCTGTCTTTGAAAAGACGATTGGAGGCAACA
sim-CG3927      AAAAATCTATAAATCTATTATTTTGCAGATCTGTCTTTGAAAAGACGATTGGAGGCAACA
                                                                     Exon4
sim-CG3875      GAACTGGGTTTCCCAAGTACAACAACCTGATTAAGCGAGATAAAGGAAATCCGCCCGA GT
sim-CG3927      GAACTGGGGTTCCCAAGTACAACAACCTGATTAAGCGAGATAAGGAAAATCCGCCCGA GT

sim-CG3875      AAGTTATCAATTTATGACC---CCACCAGGAGTTCCCGCGTCCCAGTAGTTCCTTGAATC
sim-CG3927      AAGTTATCATTTAATGACTCTACCACCAGGAGTCCCCGCGTCCCAGTAGTTCCTTGAATC
                                                                    Exon5
sim-CG3875      CTAGCCAACATGCGTTGAGCCAGCAACTAACATTTTGATCTTTATAG AATCGCCGATACG
sim-CG3927      CTAGCCAACATGCGTTGAGCCAGCAACTAACATTTTGATCTTTATAG AGTCGCCGATACG

sim-CG3875      GAGGAGGTGGCCTATAAACATCGTATGCCCCCCAGTCGTCCGTCTATGCGCTATGAGAAC
sim-CG3927      GAGGAGGTGGCCTATAAACATCGTATGCCCCCCAGTCGTCCGTCTATGCGCTTTGAGAAC
                Exon5
sim-CG3875      AGAATAC GTAAGTGTTTGGTAGTATCGGGGTCTATTACATAAGATAGTTTTATGAGATGC
sim-CG3927      AGAATAC GTAAGTGTTTGGTAGTATCGGGGTCTATTACATAAGACAGTTTTATGAGATGC

sim-CG3875      ACCTT-------TGATTCACCTT----TAT----TCAAAACGAATAACAGTGAATCAAAC
sim-CG3927      ACCTTTGAAGGGTGATTTAGATTATAATATACTATCAAAACGAATAACAGGGAATCAAAC

sim-CG3875      TATATAATACACTAAAATGATTAAAA-TATGCATCAATTTACTTCTTTGTTAATTAAAAA
sim-CG3927      TATATAATACACTATAATGATTAAAATTATACATCAATTTACTTCTTTGTTAATTAAAAA
                                                             Exon6
sim-CG3875      CGCATAAATTAATACAAATAATTATCAATCAATTTTATAG CACGTCCATCAATAATAGCG
sim-CG3927      CGCATAAATTAATACAAATAATTATCAATCAATTTTATAG CACGACCATCAATAAGAACC

sim-CG3875      ACAAAGGCTGCGCCATTTAAACGTCTATATCCGTATCCGACTGACGTGAATCGTATGCGC
sim-CG3927      ACAAACGCTGCGCCACTTAAACGTCCATATCCGTATCCGACTGACGTGAATCGTATGCGC

sim-CG3875      GAACCGCCCATCAAGTCCTATAAACCCAATCCGCATACAATACTTAAAAAGCCTATAAAT
sim-CG3927      GAACATCCCATCAAGTCCTATAAACCCAATC-----------------------------

sim-CG3875      AATAGAGCTCCGGTTGATTTTGTCGCACACCGCTATACACGAAAACAAAGGACAATGCTT
sim-CG3927      -----------------------------CTGCTATAT-----------GGA-----CTT

sim-CG3875      GCGTTATAAAATCCATGCTGGGAGCACATCGAAAATACTTTAGTCAAGAAGAAAGCAGCA
sim-CG3927      AGGTTATACAATCCG---TGGGAGCACATAAAAAATACTTAAAACACGAAGAAATCAGCA
                               Stop Codon   3'breakpoint
sim-CG3875      TTCAAAATGATGCTATCAATGAA TAA ACATTA TAAAACGCAACGCAAGTTTTTTGAACGA
sim-CG3927      TTCAAAATGATGATATCAATGAA TAA ACATTA ----------------------------
                DNAREP1_DM
sim-CG3875      TGTCCCACGCCCACTCTATTGCCAACAA
sim-CG3927      ----------------------------
```

5. kep1/CG4021

```
         5'breakpoint                                              Start Codon
mel-kep1        TTCAGTCTACACCATTTTTAATTGTAATTTTCTG-TAAAGATTGTT ATGATAAAAATGGA
```

```
mel-CG4021      TTCAGCCTACATCAATTTTAATTGTGATTTTTCGCTAAAGATTGTT ATGGAAAAAATGGA

mel-kep1        AACCCCAAGCGAGTTTACTGAGAAACAGCCACCCACC---CACGATCACCAGCCGCGTCT
mel-CG4021      AAACCCAAGCGAGATTACTGAGGAACAGCCAACTACCACACACGAGTACCAGCCGCGTCT

mel-kep1        TAACGAGGTGGCCCAAAAGTTTCTCGCCGATTTGGACGAGGAGCGCCAGCGATTGTCCGC
mel-CG4021      GAACGAGATAGCCCAAAAGTTTCTCGCCGATTTGGACGAGGAGCGCCAGCGTTTGTCCGC
                                                 Exon1
mel-kep1        GGACTTTCCACTTTGCGCACTGCTAATCGACGAGG GTAAAATCAATTTGGCCCTTTTACA
mel-CG4021      GGAGTTTCCACTTTGCGCACTGCTAATCGACGAGG GTAAAATCAATTCGTCCC-TTTACA
                                                           Exon2
mel-kep1        GGG—TATTTTAATGGTACTAATCCCAATATTGCAG CTGTGGACCGTGTCTACTGCACTG
mel-CG4021      GGGTATATTTTATTGGTACTAATCCCAA-ATTGCAG CTAGGGACCGTGTCTACGCCACTG

mel-kep1        GTCGTATTCCCGGAAAAGAGTTCTACGCAGACGTGTACAAGCAGAAGCCGATGAAGATTA
mel-CG4021      GTCGTATTCCCGGCAAGGAACTCTACGCAGACGTGTACCGGCAGAAGCCGATGAAGATTA
                                                    Exon2
mel-kep1        CCCAAAAGGTCTTCGTGCCCGTAAAGCAGTATCCCAAG GTGGG-TTTATACAATTAGCTG
mel-CG4021      TTCAAAAGGTCTTCGTGCCCGTTAACCAGTATCCCAAG GTGGGTTTTATACAATTAACTG
                                                              Exon3
mel-kep1        TCCTCTCCGTTCGAAAAATAAATCCGTACCCTTGCGCAG TTCAACTTCACTGGCAAGATC
mel-CG4021      TCCCCTATGTCCGCAAAATAAATCCGTACCCTTGCGCAG TTCAACTTCGCTGGCAAGATC

mel-kep1        CTGGGGCCCAAGGGCAATTCGCTGCGTCGCCTTCAGGAGGAGACCCAGTGCAAGATTGCC
mel-CG4021      CTGGGGCCCAAGGGGAACTCGCTGCGTCGCCTTCAGGAGGAGACCCAGTGCAAGATTGCC

mel-kep1        ATTAAGGGTCGCAGTTCGATCCGCGATCGTAACAAAGAAGAGCAGCTGCGCAGCACAGGC
mel-CG4021      CTCAAGGGTCGCAGTTCGATGCGCGATCGTAACAAAGAGGAAGAGCTGCGCA------GC

mel-kep1        GACCCGAGATATGCTCATCTTCAGAAGGACCTATTTTTGGAGGTCAGCACGGTGGCCACT
mel-CG4021      GACCCAAGATATGCCCATCTTCAGAAGAATCTCTTTCTGGAGGTCAGCACGGTGGCCATA

mel-kep1        CCGGCGGAGTGCTATGCCCGCATAGCCTACGCTCTGGCCGAGATCCGTAAGTATCTTATT
mel-CG4021      CCGGCGGAGTGCCATTCCCGCATAGCCTACGCCCTGGCCGAGATTCGTAAGTATCTGATT

mel-kep1        CCAGACAAGAACGACGAGGTTTCACACGAACAGCTGCGCGAGCTGATGGAAATGGATCCC
mel-CG4021      CCAGACAATAACGACGAGGTTTCGCACGAGCAGCTGCGCGAGCTGATGGAAATAGATCCC
                                                         Exon3
mel-kep1        GAGTCAGCCAAAAACATTCACGGACCGAATCTGGAGGCCTACAG GTA------ATCTTAC
mel-CG4021      GAGTCGGCCAAGAACTTTAAGGGACTCAATCTGGAGGCGTACAG GTAATACCGATCTGAT

mel-kep1        ACCCT-------------ATGCAGCCTTTGGGCATGA------GAATAAAAATATATTAT
mel-CG4021      ATCCTTCGCTTGCCATTAGTACAATTTTAAGTCTAGATTAATTAATTAAAAATCTATTAT
                          Exon4
mel-kep1        TTTGCAG ATCTGTCTTCGACAAGAAGTTTGGAGGCAACAGCAATGGGGCTCCCAAATACA
mel-CG4021      TTTGCAG ATCTGTC-------------------AGGGACAGCAATGGGGCTTCTAAGTACA
                                                  Exon4
mel-kep1        TCAACCTGATTAAGAGAGCTGCGGAAAATCCGCCCGA GTAAGTTACTTGTAAAGTATAAT
mel-CG4021      TCAATCTGATTAAGCGGGTTGCGGAAAATCCGTCCAA GTAAGTTACTT-----GTATAAT

mel-kep1        TTTGTATTATTTAATGACCCCACCAGGAGTAATGGAACCGAGAGTCCTCGCGTCATAGTA
mel-CG4021      TATGTATCATTTAATGACACCAATAG---------------GTATCCCCGCGTCCAAGTA
                                                                       Exon5
mel-kep1        GTGCCTTAAATCCTAGCCAACATCTGTTAACCCAGCAATTAACATTTTCATTTTTATAG A
mel-CG4021      GTTCCTTGAATCCTAG---------------TCAGCAACTAACATTTTCATCTTTATAG A

mel-kep1        GTCGACGATGTGGAGGAGGTGGCCTATGAGT---ATGAACATCGTATGCCCCCCAAGCGT
mel-CG4021      GTCGCCGATATGGAGCAGGTGGACTATGATTATGATGAACATCATATGCCCCCCATTCAT
                                     Exon5
mel-kep1        CCGCCTACGGGCTATGAGTACAGCAAAC GTAAGTTAATCCCAAGCATGCATTCTTTATTT
mel-CG4021      CTGCCCACGGGCTATGAGTACAGCATAC GTAAGT-----------GTG----------TT

mel-kep1        GGGTAGTGCC--GGGGTCTATGACATAAGATCGATTTTTCCCCTGAATGCACCAGTTTTT
mel-CG4021      TGGTAGTGCTTGGGGGTCTATTACA-----------------------------------

mel-kep1        TGGGATGCACCTTTGACAGGAACTTTGGGTTATAGATTAGATTAGATCATACTATCAAAA
mel-CG4021      ------------------------------------------------------------

mel-kep1        CCAATAACTGTGATTGAGCAAGCTCTCAGATCAAACTATATAATACACTGGAATGATTAA
mel-CG4021      --AATAA-----------------------------------------------------

mel-kep1        TATTATGCAACGATTCACTCCTTTATAGTAATTACATACACGCATAAAATACTACAAATA
```

```
mel-CG4021      --------------------------------------------------ATTACAAATA
                                      Exon6
mel-kep1        ATAATCAACCAATTTTATAG CACGTCCATCAATAATACCGACAAACGCAGCGGCATATAA
mel-CG4021      ATAATCAAGTAATTTTATAG AACGTCCATCAAAAATA---------GTTGCGAAATTTAA

mel-kep1        ACGTCCA------TATCCGACTGACATGAAACGTATGCGCGAACCGCCCATCAAGTCCTA
mel-CG4021      ACGTCCATATCCGTATCCGACTGACATGAAACCTGTGCGCGAACCGCCCATAAAGTTCTA
                                                    Stop Codon
mel-kep1        TAAGCCCAATCCGTATACAATACTTAAAAAATATAAA TAA TAGAGCTCCGGTTGATTTCG
mel-CG4021      TAAGCCCAATCCTGGTATA------------------ --- --------------------

mel-kep1        TCGCACACCGCTATAAATGAAAACAAAGGACAATGCTTACGTTATAAAATCCATGCTGGG
mel-CG4021      -------------------------------GGCACCTACGTTATGAAATCTATGCTTGG

mel-kep1        AGCACATTGAAAATACTTTAATCAAGAAGAAAGCAGCATTCAAAATGATGCTATCAATTA
mel-CG4021      AGCACAGT------------------AAGAAAGCTGCATTCAAAATGATGCTATCAATGA
                            3'breakpoint
mel-kep1        ATAAACATTAATATTTATA -----------------------------------------
mel-CG4021      AGAAACATTAACATTTATA TAAAAGCGCACATTACCGCAAACAATATTATTATAATGTAC

mel-kep1        ------------------------------------------------------------
mel-CG4021      CTTTCCTTACTTCGTAAAAAACGTTAATAAAATGATACGTATACCCAAAATAATAACTAA
                                                             DNAREP1_DM
mel-kep1        ------------------------------------------------------------
mel-CG4021      TATATAACATTTTTCAAAAGTGTTGGCGTGGCAGTTCTGGACGGTTTGGGTGCCTTGGAG

mel-kep1        ------------------------------------------------------------
mel-CG4021      TGGGCGTGGCAACATGAATTAAAAAAATTTGCGCTTCGTCTATAGTTAAACGCTTCCTTC

mel-kep1        ----------------------------------------------
mel-CG4021      TGCCTGTTACATACTTCCCAACGAACGGGTATAACTATTATTACTC
```

6. kep1/CR9337

```
sch-kep1        ------------------------------------------------------------
sch-CR9337      TTATACCCGTTACTCGTAGATTAAAAGGGTATACTAGATTCCTTTAAAAGTATGTAAAAG
                DNAREP1_DM
sch-kep1        ------------------------------------------------------------
sch-CR9337      TATATGTAAAAGTATATATATTCTTCAGAATCGATGTCTGTCCTTCCGTATGAACGTTGA

sch-kep1        ------------------------------------------------------------
sch-CR9337      GATGCCAGGAACTATAAAAGATAGAAGGTAACGATTAGGAACACAGATTCTAGAGACATA

sch-kep1        ------------------------------------------------------------
sch-CR9337      GATGCAGCGCAAGTTTGTTGAACCGTGTCCCATGCTCTAACTCTAACGCCCACAAAGCGC

sch-kep1        ------------------------------------------------------------
sch-CR9337      CCAAAACTGACACGCCCACAACACTATATCTAATTTTTCATTTTATTATTTTGTTTTCCA

sch-kep1        ------------------------------------------------------------
sch-CR9337      TTTCTACCGGTATTCCAAAATATATTAAAATATCTCGTTTGGAATAAACTGGAGAATCT

sch-kep1        ------------------------------------------------------------
sch-CR9337      CGACTATAGCATTCATGTTTTTAATCTGATGGGTAAGTAGCCTAAGTGTACCAGAGACCA

sch-kep1        ------------------------------------------------------------
sch-CR9337      TGGTGGTGTTCAGTTTTCTGCACATAATGGGATACCCGACATTTAAACAATCACTTTAGC
                           5'breakpoint          Transcription Start Position
sch-kep1        ----------------- AAAACTTCATACGGTTGAC AGCTCTGGCACCATTTTAGTTCAT
sch-CR9337      ACTGAAAAATATGCCTC AAAACTTCAAACGGTTGGC AGGTCTGCCATCATTTTAGTTCAC

sch-kep1        TGATATACATACTGTTTTTCAGTCTAC----GTTTTAATTGTAATTTTTCGGTAAGGATT
sch-CR9337      TGATATACAAAGCGTTATTCAGTCTACATCAATTTTAATTGTGATTTTTTGGTAAAGATT
                      Start Codon
sch-kep1        GTT ATG ATAAAAATGGAAACCCCAAGCGAGTTTGCTGAGAAGCAGCCTCCTACCCACGAC
sch-CR9337      GTT ACG ATAAAAATGGAAACCTCAAGCGAGTTAACTGAGAAAGAGCCACTTACCCACGAC

sch-kep1        CACCAGCCGCGTCTGAACGAGGTGGCCCAAAAGTTTCTGGCCGATTTGGACGAGGAGCGC
sch-CR9337      CACCAGCCGCGTCTGAACGAGGTGGCCCAAAAGTTTCTCGCCGATTTGGACGAGGAGCGG
                                                            Exon1
sch-kep1        CAGCGATTGTCCGCGGACTTTCCACTTTGCGCACTGCTAATCGACGAGG GTAAAATCAAT
sch-CR9337      AAGCGATTGTCCGCGGAGTTTCCACTTTGCGCACTGCTAATCGCCGAGG GTAAAATCAAT
                                                                   Exon2
```

```
sch-kep1      TCGGCTCTTTACAGGGTATATTAATGGTACTAATCCCACATTGCAG CTGTGGACCGCGTC
sch-CR9337    TCGGTCCTTTACAGGGTATTTTAATAGTACTAATCCCACATTGCAG CTGTGGACCGTGTC

sch-kep1      TACTGCACTGGTCGTATTCCCGGCAAGGAGTTCTACGCAGACGTGTACAAGCAGAAGCCG
sch-CR9337    TACGGCACTGGCCGTATTCCCGGCAAGGAGTTATACGCAGACGTGTACAACCAAAAGCCG
                                                         Exon2
sch-kep1      ATGAAGATTACACAAAAGGTATTCGTGCCCGTAAAGCAGTATCCCAAG GTGGGTTTTATA
sch-CR9337    ATGAAGATTACCCAAACGGTCTTTGTGCCCGTTAATCAGTATCCCAAG GTGGGTTTTGAA
                                                                   Exon3
sch-kep1      CAATTAACTGTCCCCTCCGTCCCCAAAATAAATCCGTATCCTTGCGCAG TTCAACTTCAC
sch-CR9337    CAATTAGCTGTCCATCCCGTCCGCAAAATGAATCCTTACCCTTGCGCAG TTCAACTTCAT

sch-kep1      TGGCAAGATCCTGGGGCCCAAGGGCAACTCGCTGCGTCGCCTTCAGGAGGAGACCCAGTG
sch-CR9337    TGGCAAGATCCTGGGGCCCAAGGGGAACTCGTTGCGTCGCCTTCAGGAGGAGACCCAGTG

sch-kep1      CAAGATTGCCATTAAGGGTCGCAGTTCGATCCGCGATCGTAACAAAGAGGAGCAGCTGCG
sch-CR9337    CAAGATTGCCATCAAGGGTCGCGGTTCGATGCGCGATCGTAACAAAGAGGAGCAGTTGCG

sch-kep1      CAGCACAGGCGATCCGAGATATGCCCATCTTCAGAAGGATCTATTCCTGGAGGTCAGCAC
sch-CR9337    CAGCACAGGTGACCCGAGATATGCCCATCTTCAGAAGAATCTCTTTCTGGAGGTCAGCAC

sch-kep1      GGTGGCCACTCCGGCGGAGTGCTATGCCCGCATAGCTTACGCCCTGGCCGAGATCCGTAA
sch-CR9337    GGTGGCCAACCCGGCGGAGTGCTATGTCCGCATAGCCTACGCCCTGGCCGAGATCCGTAA

sch-kep1      GTATCTTATTCCAGACAAGAACGACGAGGTTTCGCACGAGCAGCTGCGCGAGCTGATGGA
sch-CR9337    GTATCTTATTCCAGACAAGAACGACGCGGTTTCGCACGAGCAGCTGCGCGTGCTGAAGGA
                                                                 Exon3
sch-kep1      AATGGATCCCGAGTCGGCCAAAAACATTCACGGACCC---AATCTGGAGGCTTACAG GTA
sch-CR9337    AATGGATCCCGAGTCCGCCAAGAACAGTAACGGACTCTGGAGTCTGGAGGTCTACAG GTA

sch-kep1      AGGGGGATGTTTTACCCTATGCATACCGATGCGGTTTGCCT---------------TTGG
sch-CR9337    ATGGGGAT------CCCTCTGCATACCGATGTAACTTGACTTACTTGACAAGCGAGTTGA

sch-kep1      GCAT----------------------------AAGAATAAAAATGTA---TTATTTTGCA
sch-CR9337    GCATGTGGTATACAAAGGCTTGATATTGGGCAAGGAGTAAAAATCTATTGTTATTTTGCA
                  Exon4
sch-kep1      G ATCTGTCTTCGACAAGAAATTTGGAGGCAACAGCAATGGGGCTCCCAAGTATATCAACC
sch-CR9337    G ATCTGTCCTCGACAAGAAGTTGGGAGGCAACAGCAATGGGGCTCTCACGTACATCAACC
                                        Exon4
sch-kep1      TGATTAAGCGAGCTGCGGAAAATCCGCCCGA GTAAGTTACTTGTAAAGTATAATTTTGTA
sch-CR9337    CGATTAAGCGAGTTGCGGGAAATCCACCCGA GTAAGTTACTTGTAAAGTATTATTTTGTA

sch-kep1      TAATTTAATGACCCCACCAGGAGTTATGGACCTGAGAGTCCTCGCGTTATAGTAGTGTCA
sch-CR9337    TCATTTAATGTCC-------------TTGTTCTTTGAATCCTACC-------------CA
                                                          Exon5
sch-kep1      ATATGTGTTAACCCAGCAACTAACATTTCGATCTTTATAG AGTCGACGATGTGGAGGAGG
sch-CR9337    ACATGCGTTGAGCCAGCAACTAACATTTTGATCTTTATAG AGTCGACGATGTGGAGGAGG

sch-kep1      TGGCCTATGAGTATGAACATCGTATGCCCCCCAAGCGTCCTCCTACTGGCTATGAGTACA
sch-CR9337    TGGTCTATG---------ATCTTATGCCCCCCAGTCATCCGCCTACGAGCTATGAGTACA
              Exon5
sch-kep1      GCAAAC GTAAGTTGATCCCAAGCATGCATTCTTTATTTGGGTAGTGCCGGGGTCTATAAA
sch-CR9337    GGAAAC GTAAG----------------------TATTT-GGTAGTGCCGGGGTCCAT-TA

sch-kep1      CATAATATAGATTTCCCCCTAAAGGCACTTCGCCAGTTTTTGGGGATTTGGGTTATAGAT
sch-CR9337    CATAAGATTGTTTT---ATGGGATGCATCT------TTGACGGGAACTTGGGATTTAGAT

sch-kep1      TAGATCATACTATCAA-----AACCAATAACAGTGATTGAGCAAGCTCTCAGATCAAACT
sch-CR9337    ACGAT--TATTATCGAGTATTAACTGACAACAGTGAATGATCAAGATCTCAGATCAAACT

sch-kep1      ATATAATACACTGGAATGAT--------TAATATTATGCAACGATTCACTTCTTTATAGT
sch-CR9337    ATATAATACACTACAATGACTAATATTATAATATTATACAACGATTAACTTCTTTATAGT
                                                                     Exon6
sch-kep1      AATTACATACACGCATAACTTACTACAAATAATAATCAACCAATTTTATAG CACGTCCAT
sch-CR9337    AATTACATACACGCATAAATTACTACTAATAATAATCAACCAATTTTATAG CATTTCCAT

sch-kep1      CAATAATACCGACAAACGCTGCGGCATATAAACGTCCATATCCG------ACTGACATGA
sch-CR9337    TAAAAATACCGGAAAACGCAGTGGCATATAAACGTCCATATCCGTATCTTACTGACATGA

sch-kep1      AACGTATGCGCGAACCGCCCATCAAGTCCTATAAGCCCAATCCGTATACAATACTTAAAA
sch-CR9337    AATTTATGCGCGAACCGCCCATCAAGAACTTTAAGCCCAATCCTTTTA--------AACG
              Stop Codon
```

```
sch-kep1        AATATAAA TAA TAGAGCTCCGGTTGATTTCGTCGCACACCGCTATAAATGAAAACAAAGG
sch-CR9337      GACTTAAG TTA -------------------------------------------------

sch-kep1        ACAATGCTTAAAATCCATGCTGGGAGCACATTGAAAATACTTTAATCAAGAAGAAAGCAG
sch-CR9337      --------TAAAATCCATGATGGGAGCACATTGAAAATACTTTAAACAAGATGGAAGCAG

sch-kep1        CATTCAAAATGATGCTATCAATGAATAAACATTAACATTTATAAAACGCGCACTTGCGCT
sch-CR9337      ATTTCAA------------AATGAATAAATATTAACATTTATAAAAACCGCACTTGCGCT

sch-kep1        ATCAATTTCCACAAACAATATTAACCTTAAATTTTTATTCCCATAAATATGTTAAACTGT
sch-CR9337      ATCAAATCCTACAAACAATATTAATCTTAATTATTTATTTCCCTAATTATGTTGAGCTGT

sch-kep1        TGGCATGCCTTTTCTGGACCATTTCAACAGATAATAAGTCAAATAATTCCTATTATGTTT
sch-CR9337      TGGCATG-----------------------------------------CCCATTATGTTT

sch-kep1        AAATTATGATTTCCTGAGTTCGTAATTTC---TTATGAATTAAAAAAATGTAATAAAAGG
sch-CR9337      AAATTAAAA--TCCTGCGTTTGTAATTTTCTATTATAAATTAAAACAC--TAATAAAATA
                                  Polyadenylation Site
sch-kep1        ATACGCATTGAATGAATTTAATAAATTCTCAGCAATCAGA TCTAACTATGCCCGATTCTT
sch-CR9337      AAACTCATTGAAT--ATTTTATAAATT-----CAATCCGA TCTAATTATGCCCG------

sch-kep1        TTAAATGACTCGACCCCCTCATTTATAATGAATA--TGCATTACGTAATTAAACAATTGT
sch-CR9337      --------------------ATTTACAATCAATATTTGCATTACCTAATTAAACAATTAA

sch-kep1        CTCACTAGCAGTAGAATCAAG--AGTTATATATCTAATAAAATAAAACGGTACGAACTTA
sch-CR9337      CATACTAGCATTAAAAGTTAGCTAGTTATATTTCTAA-AAAATGAATTGA----------

sch-kep1        TCACATTGTTTAATCAAAAAAAAAAAAAACGAAATGAAGATTACATCAAGACATTTAAGTG
sch-CR9337      --ACATCCTTTTATC-----AGATGAATCAAAGTGAAGATCGCATCAAGATACTTAAGTG

sch-kep1        CGTCTAAAAATAAGTTAATAAACAATTATAAATTTATAAATGAACAAAAAAAGAACATTG
sch-CR9337      CTTTTAAAAATAATTTCATAATCCATCATAATTTTATAAATGAAC-AAGAGAGAACAATA
                                                       3'breakpoint
sch-kep1        AATAAACATATAAAGAGTTTGCATAACTAAAATATTTCAGGTTTTTT -------------
sch-CR9337      AATAAACATATAAAGAGTTTGCATAACTAAAATATTTCAGGTTTTTT AGGCAAAGTATTA

sch-kep1        ------------------------------------------------------------
sch-CR9337      ATTTGTATACCCGTTAGTCGCAAAGTAAAAGGGTCTACCAGATTCGTTGAAAACTATGTA
                     DNAREP1_DM
sch-kep1        ------------------------------------------------------------
sch-CR9337      ACTGGTAGAAGAAAGCGTTTGTTGACCCATATTATCATGCCCACTCCAACGCCCACAAAC

sch-kep1        ------------------------------------------------------------
sch-CR9337      CGCACAAATCTGTCATGCCTAGACTTTAGAAAAATGTGTTGATATTTTTTTCATAATTTG

sch-kep1        ------------------------------------------------------------
sch-CR9337      CAAAAAACTGTTTGCCACTCCCAGTCTAACGCCCTAAAGTCGCCGAATGCCCACACTTTT

sch-kep1        ------------------------------------------------------------
sch-CR9337      GAACAATTTTAAAAATTTTTTCCAATTTTATTCCGCAATATTTATCGATATACCAGAAAA

sch-kep1        ------------------------------------------------------------
sch-CR9337      ATTACGAAATTCGCGTTTTGCATTCATACTAGCTGAGTAACGGGTAACTTGCTATAGCAT

sch-kep1        -------------
sch-CR9337      TTCTCTCTTGCTT
```

7. CR9337/CR33318

```
                 DNAREP1_DM
sch-CR9337       TTATACCCGTTACTCGTAGATTAAAAGGGTATACTAGATTCCTTTAAAAGTATGTAAAAG
mel-CR33318      ------------------------------------------------------------

sch-CR9337       TATATGTAAAAGTATATATATTCTTCAGAATCGATGTCTGTCCTTCCGTATGAACGTTGA
mel-CR33318      ------------------------------------------------------------

sch-CR9337       GATGCCAGGAACTATAAAAGATAGAAGGTAACGATTAGGAACACAGATTCTAGAGACATA
mel-CR33318      ------------------------------------------------------------

sch-CR9337       GATGCAGCGCAAGTTTGTTGAACCGTGTCCCATGCTCTAACTCTAACGCCCACAAAGCGC
mel-CR33318      --------------------AACTTTTTGCCACGCCC--ACTCTAACGCCC-TAAAGTCG
                                     DNAREP1_DM
sch-CR9337       CCAAAACTGACACGCCCACAACACTATATCTAATTTTTCATTTTATTATTTTGTTTTCC-
```

```
mel-CR33318      CCAAACCTTGCACGTCCAC-ACTTTAGAAC-AATTTTT--TTTATTTATTTTATTACCCA

sch-CR9337       ATTTCTACCGGTATTCCAAAATA--TATTAAAATATCTCGTTT-------------TGGA
mel-CR33318      ATATCTACCGATATCCCAGAAAAATTATGAAACTTCCGCGTTTGCATTCACACTAGCTGA

sch-CR9337       ATA-------------AACTGGAGAATCTCGACTATAGCAT----TCATGTTTTTAATCT
mel-CR33318      GTAACGGTTACCTGATAGTCGGGGAACATAGACTATAGCATTATCTCTTGTTTTTAATCT

sch-CR9337       GATG---------------GGTAAGTAGCCTAAGTGTACCAGAG----------------
mel-CR33318      GATGTAGCTTAAGTATGACTGTAAATGTCTAAAGTAAGGCTGAGTTAATAAACGCTTAAA

sch-CR9337       -----------------------------------------------------ACCATGG-
mel-CR33318      ACAAATGTATGTATGTTCATCTTTGGATATACATATTTGCTTTTGGGGAACAACTACGGA

sch-CR9337       ----------------TGGTGTTCAGTTTTCTGCAC---------ATAATGGGATACCCG
mel-CR33318      TATGGTTATCTTTCCACGGGGTTCTATTTTTTATATTTTTAACTAAGGATTGGCGACTGG
                    5'breakpoint
sch-CR9337       ACATTTAA CAATCACTTTAGCACTGAAAAATAT----GCCTCAAAACTTCAAACGGTTG
mel-CR33318      ACCGTTGTT CAATCACTATAGCACTGAAAAAGTTGGCAGCCTCAAAACTTCAAACTTTTG

sch-CR9337       GCAGGTCTGCCATCATTTTAGTTCACTGATATACAAAGCGTTATTCAGTCTACATCAATT
mel-CR33318      GCAGGTCTCC---CTTTTTAAGTCACTGATATATAATTCTTTATTCAGTCTACATCAATT
                                                             Start Codon
sch-CR9337       TTAATTGTGATTTTTTGGTAAAGATTGTTACGATAAAA ATG GAAACCTCAAGCGAGTTAA
mel-CR33318      TCAATTGTGATTTTTCGGTAAATATTGTTACGATAAAA ATG GGAACAGCAAGCGCGTTAC

sch-CR9337       CTGAGAAAGAGCCACTTACCCACGACCACCAGCCGCGTCTGAACGAGGTGGCCCAAAAGT
mel-CR33318      CTTTGAAACAGCCACCTTCCCACGACCACCAGCCGCGTCTGAACGAGGTGACCCAAAAGT

sch-CR9337       TTCTCGCCGATTTGGACGAGGAGCGGAAGCGATTGTCCGCGGAGTTTCCACTTTGCGCAC
mel-CR33318      TTCTCGCCGATTTGGTCGAGGAGAGCCAGTGATTGTCCGCGGAGTTTCCACTTTGCGCAC
                         Exon1
sch-CR9337       TGCTAATCGCCGAGG GTAAAATCAATTCGGTCCTTTACAGGGTATTTTAATAGTACTAAT
mel-CR33318      TGCTAATTGCCGAGG GTAAAATCAATTCGGCCCTTTACAGGGTATTTTAATGGTATTAAT
                                  Exon2
sch-CR9337       CCCACATTGCAG CTGTGGACCGTGTCTACGGCACTGGCCGTATTCCCGGCAAGGAGTTAT
mel-CR33318      CCCACATTGCAG CTGTGGACCGTGTCTATGGCACTGGACGTATTCCCGGCAAGGAGTTAT

sch-CR9337       ACGCAGACGTGTACAACCAAAAGCCGATGAAGATTACCCAAACGGTCTTTGTGCCCGTTA
mel-CR33318      ACGCAGACGTGTACAAGCAGAAGCCGATAAAGATTACCCAAAAGGTCTTCGTGCCCGTTA
                         Exon2
sch-CR9337       ATCAGTATCCCAAG GTGGGTTTTGA-ACAATTAGCTGTCCATCCCGTCCGCAAAATGAAT
mel-CR33318      ATCAGTATCCCAAG GTGGTTTTTTATACAATTAGCTATCCACTCCGACCGCAAAATGAAT
                                       Exon3
sch-CR9337       CCTTACCCTTGCGCAG TTCAACTTCATTGGCAAGATCCTGGGGCCCAAGGGGAACTCGTT
mel-CR33318      CCTTACACTTGCGCAG TTCAACTTCACTGGCAAGATCCTGGGGACCAAGGGGAAT-----

sch-CR9337       GCGTCGCCTTCAGGAGGAGACCCAGTGCAAGATTGCCATCAAGGGTCGCGGTTCGATGCG
mel-CR33318      ------------------------------------------------------------

sch-CR9337       CGATCGTAACAAAGAGGAGCAGTTGCGCAGCACAGGTGACCCGAGATATGCCCATCTTCA
mel-CR33318      ------------------------------------------------------------

sch-CR9337       GAAGAATCTCTTTCTGGAGGTCAGCACGGTGGCCAACCCGGCGGAGTGCTATGTCCGCAT
mel-CR33318      ------------------------------------------------------------

sch-CR9337       AGCCTACGCCCTGGCCGAGATCCGTAAGTATCTTATTCCAGACAAGAACGACGCGGTTTC
mel-CR33318      ------------------------------------------------------------

sch-CR9337       GCACGAGCAGCTGCGCGTGCTGAAGGAAATGGATCCCGAGTCCGCCAAGAACAGTAACGG
mel-CR33318      ------------------------------------------------------------
                                    Exon3
sch-CR9337       ACTCTGGAGTCTGGAGGTCTACAG GTAATGGGGATCCCTCTGCATACCGATGTAACTTGA
mel-CR33318      ------------------------ -------------------------------------

sch-CR9337       CTTACTTGACAAGCGAGTTGAGCATGTGGTATACAAAGGCTTGATATTGGGCAAGGAGTA
mel-CR33318      ------------------------------------------------------------
                                           Exon4
sch-CR9337       AAAATCTATTGTTATTTTGCAG ATCTGTCCTCGACAAGAAGTTGGGAGGCAACAGCAATG
mel-CR33318      ---------------------- ---------------------------------------
                                                                 Exon4
sch-CR9337       GGGCTCTCACGTACATCAACCCGATTAAGCGAGTTGCGGGAAATCCACCCGA GTAAGTTA
```

```
mel-CR33318       --GCTCT--------------------------------------------- --------

sch-CR9337        CTTGTAAAGTATTATTTTGTATCATTTAATGTCCTTGTTCTTTGAATCCTACCCAACATG
mel-CR33318       ------------------------------------------------------------
                                                        Exon5
sch-CR9337        CGTTGAGCCAGCAACTAACATTTTGATCTTTATAG AGTCGACGATGTGGAGGAGGTGGTC
mel-CR33318       ----------------------------------- -------------------------
                                                                   Exon5
sch-CR9337        TATGATCTTATGCCCCCCAGTCATCCGCCTACGAGCTATGAGTACAGGAAAC GTAAGTAT
mel-CR33318       ---------------------------------------------------- --------

sch-CR9337        TTGGTAGTGCCGGGGTCCATTACATAAGATTGTTTTATGGGATGCATCTTTGACGGGAAC
mel-CR33318       ------------------------------------------------------------

sch-CR9337        TTGGGATTTAGATACGATTATTATCGAGTATTAACTGACAACAGTGAATGATCAAGATCT
mel-CR33318       ------------------------------------------------------------

sch-CR9337        CAGATCAAACTATATAATACACTACAATGACTAATATTATAATATTATACAACGATTAAC
mel-CR33318       ------------------------------------------------------------

sch-CR9337        TTCTTTATAGTAATTACATACACGCATAAATTACTACTAATAATAATCAACCAATTTTAT
mel-CR33318       ------------------------------------------------------------
                       Exon6
sch-CR9337        AG CATTTCCATTAAAAATACCGGAAAACGCAGTGGCATATAAACGTCCATATCCGTATCT
mel-CR33318       -- ----------------------------------------------------------

sch-CR9337        TACTGACATGAAATTTATGCGCGAACCGCCCATCAAGAACTTTAAGCCCAATCCTTTTAA
mel-CR33318       ------------------------------------------------------------
                 Stop Codon
sch-CR9337        ACGGACT TAA GTTATAAAATCCATGATGGGAGCACATTGAAAATACTTTAAACAAGATGG
mel-CR33318       ------- --- --------------------------------------------------

sch-CR9337        AAGCAGATTTCAAAATGAATAAATATTAACATTTATAAAAACCGCACTTGCGCTATCAAA
mel-CR33318       ----------------------------------------------CTTGCGCTATAAAT

sch-CR9337        TCCTACAAACAATATTAATCTTAATTATTTATTTCCCTAATTATGTTGAGCTGTTGGCAT
mel-CR33318       TCCCACAACAAATATTAATCTTAATTTTTGATTT----------------------GCAT

sch-CR9337        GCCCATTATG-TTTAAATTAAAATCCTGCGTTTGTAATTTTCTATTATAAATTAAAACAC
mel-CR33318       GCCTATTATGTTTTGATTTTGATTCCTGAGCTTGTAATTTTCTATGATAAATTCAAAAAC

sch-CR9337        TAATAAAATAAAACTCATTGAAT--ATTTTATAAATT-----CAATCCGATCTAATTATG
mel-CR33318       TAATGAAATAAAACGCATTGAATGAATTTTATAAATTTTTTGCAATCCGATCTAACTATG

sch-CR9337        CCCGATTTACAATCAATATTTGCATTACCTAATTAAACAATTAACATACTAGCATTAAAA
mel-CR33318       CCCGATATACAATCAAAATATGCATTACCTAATTAAACAATTAACTTACTAGCGTTAAAA

sch-CR9337        GTTAGCTAGTTATATTTCTAAAAAATGAATTGA---------------------------
mel-CR33318       GTTAGTTAGTTATATATCTAAAAAATAAATTGATAATGAAATAAAGCGGTATGAACTTAT

sch-CR9337        -ACATCCTTTTATCAGATGAATCAAAGTGAAGATCGCATCAAGATACTTAAGTGCTTTTA
mel-CR33318       CACATCCTTTTATCAAATGAAGCAAAGTGAA----------------------ACGATTC
                                                    3’breakpoint
sch-CR9337        AAAATAATTTCATAATCCATCATAATTTTATAAATGAACAAGA GAGAACAATAAATAAAC
mel-CR33318       AAAAAAACTTTATAATCCATTACAATTTTATAAATGAACAAGA -----------------

sch-CR9337        ATATAAAGAGTTTGCATAACTAAAATATTTCAGGTTTTTTAGGCAAAGTATTAATTTGTA
mel-CR33318       ------------------------------------------------------------
                  DNAREP1_DM
sch-CR9337        TACCCGTTAGTCGCAAAGTAAAAGGGTCTACCAGATTCGTTGAAAACTATGTAACTGGTA
mel-CR33318       ------------------------------------------------------------

sch-CR9337        GAAGAAAGCGTTTGTTGACCCATATTATCATGCCCACTCCAACGCCCACAAACCGCACAA
mel-CR33318       ------------------------------------------------------------

sch-CR9337        ATCTGTCATGCCTAGACTTTAGAAAAATGTGTTGATATTTTTTTCATAATTTGCAAAAAA
mel-CR33318       ------------------------------------------------------------

sch-CR9337        CTGTTTGCCACTCCCAGTCTAACGCCCTAAAGTCGCCGAATGCCCACACTTTTGAACAAT
mel-CR33318       ------------------------------------------------------------

sch-CR9337        TTTAAAAATTTTTTCCAATTTTATTCCGCAATATTTATCGATATACCAGAAAAATTACGA
```

```
mel-CR33318       ------------------------------------------------------------

sch-CR9337        AATTCGCGTTTTGCATTCATACTAGCTGAGTAACGGGTAACTTGCTATAGCATTTCTCTC
mel-CR33318       ------------------------------------------------------------

sch-CR9337        TTGCTT
mel-CR33318       ------
```

8. CR9337/CR9337-r

```
                5'breakpoint
sch-CR9337         TTATACCCGTTACTCGTAGATTAAAAGGGTATACTAGATTCCTTTAAAAGTATGTAAAAG
sch-CR9337-r       TTATACCCGTTACTCGCAGAGTAAAAGGGTATACTAGATTCGTTGAAAAGTATGTAACAG
                   DNAREP1_DM
sch-CR9337         ---------------------TATATGTAAAAGTATATATATTCTTCAGAATCGATGTCT
sch-CR9337-r       ACAGAAGAGAGCGTTCCCGACCATAT---AAAGTATATATATTCTTCAGAATCGATGTCT

sch-CR9337         GTCCTTCCGTATGAACGTTGAGATGCCAGGAACTATAAAAGATAGAAGGTAACGATTAGG
sch-CR9337-r       GTCCTTCCGTATGAACGTTGAGATGCCAGGAACTATAAAAGATAGAAGGTAACGATTAGG

sch-CR9337         AACACAGATTCTAGAGACATAGATGCAGCGCAAGTTTGTTGAACCGTGTCCCATGCTCTA
sch-CR9337-r       AACACAGATTCTAGAGACATAGATGCAGCGCAAGTTTGTTGAACCGTGTCCCATGCTCTA

sch-CR9337         ACTCTAACGCCCACAAAGCGCCCAAAACTGACACGCCCACAACACTATATCTAATTTTTC
sch-CR9337-r       ACTCTAACGCCCACAAAGCGCCCAAAACTGACACGCCCACAACACTATATCTAATTTTTC

sch-CR9337         ATTTTATTATTTTGTTTTCCATTTCTACCGGTATTCCAAAATATATTAAAATATCTCGTT
sch-CR9337-r       ATTTTATTATTTTGTTTTCCATTTCTACCGGTATTCCAAAATATATTAAAATATCTCGTT

sch-CR9337         TTGGAATAAACTGGAGAATCTCGACTATAGCATTCATGTTTTTAATCTGATGGGTAAGTA
sch-CR9337-r       TTGGAATAAACTGGAGAATCTCGACTATAGCATTCATGTTTTTAATCTGATGGGTAAGTA

sch-CR9337         GCCTAAGTGTACCAGAGACCATGGTGGTGTTCAGTTTTCTGCACATAATGGGATACCCGA
sch-CR9337-r       GCCTAAGTGTACCAGAGACCATGGTGGTGTTCAGTTTTCTGCACATAATGGGATACCCGA

sch-CR9337         CATTTAAACAATCACTTTAGCACTGAAAAATATGCCTCAAAACTTCAAACGGTTGGCAGG
sch-CR9337-r       CATTTAAACAATCACTTTAGCACTGAAAAATATGCCTCAAAACTTCAATCGGTTGGCAGG

sch-CR9337         TCTGCCATCATTTTAGTTCACTGATATACAAAGCGTTATTCAGTCTACATCAATTTTAAT
sch-CR9337-r       TCTGCCATCATTTTAGTTCACTGATATACAAAGCGTTATTCAGTCTACATCAATTTTAAT
                                                   Start Codon
sch-CR9337         TGTGATTTTTTGGTAAAGATTGTTACGATAAAA ATG GAAACCTCAAGCGAGTTAACTGAG
sch-CR9337-r       TGTGATTTTTTGGTAAAGATTGTTACGATAAAA ATG GAAACCTCAAGCGAGTTAACTGAG

sch-CR9337         AAAGAGCCACTTACCCACGACCACCAGCCGCGTCTGAACGAGGTGGCCCAAAAGTTTCTC
sch-CR9337-r       AAAGAGCCACTTACCCACGACCACCAGCCGCGTCTGAACGAGGTGGCCCAAAAGTTTCTC

sch-CR9337         GCCGATTTGGACGAGGAGCGGAAGCGATTGTCCGCGGAGTTTCCACTTTGCGCACTGCTA
sch-CR9337-r       GCCGATTTGGACGAGGAGCGGAAGCGATTGTCCGCGGAGTTTCCACTTTGCGCACTGCTA
                      Exon1
sch-CR9337         ATCGCCGAGG GTAAAATCAATTCGGTCCTTTACAGGGTATTTTAATAGTACTAATCCCAC
sch-CR9337-r       ATCGCCGAGG GTAAAATCAATTCGGCCCTTTACAGGGTATTTTAATAGTACTAATCCCAC

                           Exon2
sch-CR9337         ATTGCAG CTGTGGACCGTGTCTACGGCACTGGCCGTATTCCCGGCAAGGAGTTATACGCA
sch-CR9337-r       ATTGCAG CTGTGGACCGTGTCTACAGCACTGGTCGTATTCCCGGCAAGGAGTTATACGCA

sch-CR9337         GACGTGTACAACCAAAAGCCGATGAAGATTACCCAAACGGTCTTTGTGCCCGTTAATCAG
sch-CR9337-r       GACGTGTACAACCAAAAGCCGATGAAGATTACCCAAACGGTCTTTGTGCCCGTTAATCAG
                     Exon2
sch-CR9337         TATCCCAAG GTGGGTTTTGAACAATTAGCTGTCCATCCCGTCCGCAAAATGAATCCTTAC
sch-CR9337-r       TATCCCAAG GTGGGTTTTGAACAATTAGCTGTCCATCCCGTCCGCAAAATGAATCCTTAC
                               Exon3
sch-CR9337         CCTTGCGCAG TTCAACTTCATTGGCAAGATCCTGGGGCCCAAGGGGAACTCGTTGCGTCG
sch-CR9337-r       CCTTGCGCAG TTCAACTTCATTGGCAAGATCCTGGGGCCCAAGGGGAACTCGTTGCGTCG

sch-CR9337         CCTTCAGGAGGAGACCCAGTGCAAGATTGCCATCAAGGGTCGCGGTTCGATGCGCGATCG
sch-CR9337-r       CCTTCAGGAGGAGACCCAGTGCAAGATTGCCATCAAGGGTCGCGGTTCGATGCGCGATCG

sch-CR9337         TAACAAAGAGGAGCAGTTGCGCAGCACAGGTGACCCGAGATATGCCCATCTTCAGAAGAA
sch-CR9337-r       TAACAAAGAGGAGCAGTTGCGCAGCACAGGTGACCCGAGATATGCCCATCTTCAGAAGAA

sch-CR9337         TCTCTTTCTGGAGGTCAGCACGGTGGCCAACCCGGCGGAGTGCTATGTCCGCATAGCCTA
```

```
sch-CR9337-r      TCTCTTTCTGGAGGTCAGCACGGTGGCCAACCCGGCGGAGTGCTATGTCCGCATAGCCTA

sch-CR9337        CGCCCTGGCCGAGATCCGTAAGTATCTTATTCCAGACAAGAACGACGCGGTTTCGCACGA
sch-CR9337-r      CGCCCTGGCCGAGATCCGTGAGTATCTTATTCCAGACAAGAACGACGCGGTTTCGCACGA

sch-CR9337        GCAGCTGCGCGTGCTGAAGGAAATGGATCCCGAGTCCGCCAAGAACAGTAACGGACTCTG
sch-CR9337-r      GCAGCTGCGCGAGCTGAAGGAAATGGAACCCGAGTCGGCCAAGAACAGTAACGGACTCTG
                              Exon3
sch-CR9337        GAGTCTGGAGGTCTACAG GTAATGGGGATCCCTCTGCATACCGATGTAACTTGACTTACT
sch-CR9337-r      GAGTCTGGAGGTCTACAG GTAATGGGGATCCCTCTGCATACCGATGTAACTTGACTTACT

sch-CR9337        TGACAAGCGAGTTGAGCATGTGGTATACAAAGGCTTGATATTGGGCAAGGAGTAAAAATC
sch-CR9337-r      TGACAAGCGAGTTGAGCATGTGGTATACAAAGGCTTGATATTGGGCAAGGAGTAAAAATC
                                      Exon4
sch-CR9337        TATTGTTATTTTGCAG ATCTGTCCTCGACAAGAAGTTGGGAGGCAACAGCAATGGGGCTC
sch-CR9337-r      TATTGTTATTTTGCAG ATCTGTCCTCGACAAGAAGTTGGGAGGCAACAGCAATGGGGCTC
                                                          Exon4
sch-CR9337        TCACGTACATCAACCCGATTAAGCGAGTTGCGGGAAATCCACCCGA GTAAGTTACTTGTA
sch-CR9337-r      TCACGTACATCAACCCGATTAAGCGAGTTGCGGGAAATCCACCCGA GTAAGTTACTTGTA

sch-CR9337        AAGTATTATTTTGTATCATTTAATGTCCTTGTTCTTTGAATCCTACCCAACATGCGTTGA
sch-CR9337-r      AAGTATTATTTTGTATCATTTAATGTCCTTGTTCTTTGAATCCTACCCAACATGCGTTGA
                                                  Exon5
sch-CR9337        GCCAGCAACTAACATTTTGATCTTTATAG AGTCGACGATGTGGAGGAGGTGGTCTATGAT
sch-CR9337-r      GCCAGCAACTAACATTTTGATCTTTATAG AGTCGACGATGTGGAGGAGGTGGCCTATGAT
                                                          Exon5
sch-CR9337        CTTATGCCCCCCAGTCATCCGCCTACGAGCTATGAGTACAGGAAAC GTAAGTATTTGGTA
sch-CR9337-r      CTTATGCCCCCCAGTCATCCGCCTACGAGCTATGAGTACAGGAAAC GTAAGTATTTGGTA

sch-CR9337        GTGCCGGGGTCCATTACATAAGATTGTTTTATGGGATGCATCTTTGACGGGAACTTGGGA
sch-CR9337-r      GTGCCGTGGTCCATTACATAAGATTGTTTTATGGGATGCATCTTTGACGGGAACTTGGGA

sch-CR9337        TTTAGATACGATTATTATCGAGTATTAACTGACAACAGTGAATGATCAAGATCTCAGATC
sch-CR9337-r      TTTAGATACGATTATTATCGAGTATTAACTGACAACAGTGAATGAGCAAGATCTCAGATC

sch-CR9337        AAACTATATAATACACTACAATGACTAATATTATAATATTATACAACGATTAACTTCTTT
sch-CR9337-r      AAACTATATAATACACTACAATGACTAATATTATAATATTATACAACGATTAACTTCTTT
                                                                          Exon6
sch-CR9337        ATAGTAATTACATACACGCATAAATTACTACTAATAATAATCAACCAATTTTATAG CATT
sch-CR9337-r      ATAGTAATTACATACACGCATAAATTACTACTAATAATAATCAACCAATTTTATAG TATT

sch-CR9337        TCCATTAAAAATACCGGAAAACGCAGTGGCATATAAACGTCCATATCCGTATCTTACTGA
sch-CR9337-r      TCCATTAAAAATACCGGAAAACGCAGTGGCATATAAACGTCCATATCCGTATCTTACTGA

sch-CR9337        CATGAAATTTATGCGCGAACCGCCCATCAAGAACTTTAAGCCCAATCCTTTTAAACGGAC
sch-CR9337-r      CATGAAATTTATGCGCGAACCGCCCATCAAGAACTTTAAGCCCAATCCTTTTAAACGGAC
            Stop Codon
sch-CR9337        T TAA GTTATAAAATCCATGATGGGAGCACATTGAAAATACTTTAAACAAGATGGAAGCAG
sch-CR9337-r      T TAA GTTATAAAATCCATGATGGGAGCACATTGAAAATACTTTAAACCAGATGGAAGCAG

sch-CR9337        ATTTCAAAATGAATAAATATTAACATTTATAAAAACCGCACTTGCGCTATCAAATCCTAC
sch-CR9337-r      ATTTCAAAATGAATAAATATTAACATTTATAAAAACCCCACTTGCGCTATCAATTCCTAC

sch-CR9337        AAACAATATTAATCTTAATTATTTATTTCCCTAATTATGTTGAGCTGTTGGCATGCCCAT
sch-CR9337-r      AAACAATATTAATCTTAATTATTTATTTCCCTAATTATGTTGAGCTGTTGGCATGCCCAT

sch-CR9337        TATGTTTAAATTA--AAATCCTGCGTTTGTAATTTTCTATTATAAATTAAAACACTAATA
sch-CR9337-r      TATGTTTAAATTATGATATCCTGCGTTTGTAATTTTCTATTATAAATTAAAAAACTAATA

sch-CR9337        AAATAAAACTCATTGAATATTTTATAAATTCAATCCGATCTAATTATGCCCGATTTACAA
sch-CR9337-r      AAATAAAACTCATTGAATATTTTATAAATTCAATCCGATCTAACTATGCCCGATTTACAA

sch-CR9337        TCAATATTTGCATTACCTAATTAAACAATTAACATACTAGCATTAAAAGTTAGCTAGTTA
sch-CR9337-r      TCAATATATGCATTACCTAAATAAACAATTAACA-ACTAGCATTAAAAGTTGGTTAGTTA

sch-CR9337        TATTTCTAAAAAATGAATTGA----------------------------ACATCCTTTTA
sch-CR9337-r      TATATCT-AAAAATAAATTGGTAATTACATAAAACGGTACGAACTTATCACATCCTTTTA

sch-CR9337        TCAGATGAATCAAAGTGAAGATCGCATCAAGATACTTAAGTGCTTTTAAAAATAATTTCA
sch-CR9337-r      TTAAATGAAGCGACGTGAAGATCGCATCAAGACATTTAAGTGCTCTTAAAAATAATTTAA

sch-CR9337        TAATCCATCATAATTTTATAAATGAACAAGAGAGAACAATAAATAAACATATAAAGAGTT
```

```
sch-CR9337-r      TAAACCATTATAATTTTATAAATGAACACGAAAGAACATTAAATAAA-------------
                                                              DNAREP1_DM
sch-CR9337        TGCATAACTAAAATATTTCAGGTTTTTTAGGCAAAGTATTAATTTGTATACCCGTTAGTC
sch-CR9337-r      ---ATAACTAAAATATTTCAGG-TTTTTAAGCAAAGTATTCATTTTTATACCCGTTA---
                                                                  3'breakpoint
sch-CR9337        GCAAAGTAAAAGGGTCTACCAGATTCGTTGAAAACTATGTAACTGGTAGAAGAAAGCG T
sch-CR9337-r      -------AAGAGGGTATATAAGATTCGTTGAAAACTATTTAACAGGTAGAAGGAAGCGT C

sch-CR9337        T------------------------------GTTGACCC---------------------
sch-CR9337-r      TCCGACCATATTGATCAGAATCAATAGCCGAGTCGATCTAGCCACATCTGTCCGCCTGCC

sch-CR9337        ----------------------------------------------ATATTATCATGCCC
sch-CR9337-r      TTCTGTCCGTATGCACGTCGAGATCTCAGGAACTAGAAAAGCTAGAAGGTTGCCACGCCC

sch-CR9337        ACTCCAACGCCCACAAACCGCACAAATCTGTCATGCCTAGACTTTAGAAAAATGTGTTGA
sch-CR9337-r      ACTCAAACGCCAACAAACCGC-CATACCTGCCACGCCAACATCGTTGAAGAATTTGTGG-

sch-CR9337        TATTTTTTTCATAATTTGCAAAAAACTGT-TTGCCACTCCCAGTCTAACGCCCTAAAGTC
sch-CR9337-r      -TGTTTTTTCTTAATCT-----AATCCGTCTTGTAAATT----TCTA------TACATTC

sch-CR9337        GCC-------GAATGCCCACACTTTTGAACAATTTTAAA---AATTTTTTCCAATTTTAT
sch-CR9337-r      GCCAAAAAAAAAAAGCATACACTTCTGAAAAA----AAATGTAATTTTTTCTCATTTTAT

sch-CR9337        TCCGCAATATTTATCGATATACCAGAAAAATTACGAAATTCGCGTTTTGCATTCATACTA
sch-CR9337-r      TTCCCAA----TATCCAAATCCCAGAAAAATGATGAAA------TTTCGCATTCACACTT

sch-CR9337        GCTGAGTAACGGGTAACTTGCTATAGCATTTCTCTCTTGCTT
sch-CR9337-r      GCTGAGTAACGGGTA---------------------------
```

9. CG7692/CG9902

```
mel-CG7692      ------------------------------------------------------------
mel-CG9902      AACAAGAGAGCATGCTATAGTCGAATTCCCCGACTATCAGATACCCGAAAAGAGTTGTTT
                DNAREP1_DM
mel-CG7692      ------------------------------------------------------------
mel-CG9902      GGCAAATGGATAGAAATTTAAGAGACTAATAAAAATATGAAAAAATTCAAAACTTTTTTT

mel-CG7692      ------------------------------------------------------------
mel-CG9902      TAAAAGTGAGGGCATGCCCGTTTTGGGCGGTTTGTGGGCGCGGCAACATGGGTCAACAAA

mel-CG7692      ------------------------------------------------------------
mel-CG9902      CAGTTCTTGAGATCTCGACTTTCATGCGGACGGACACGTCCAGATTGACTCGGCTATTGG

mel-CG7692      ------------------------------------------------------------
mel-CG9902      TCCTGATCAAGAATATATATACTTTTATATGGTCGGAAACGCTATTGATCGTGATCAAGA

mel-CG7692      ------------------------------------------------------------
mel-CG9902      ATATATATACTTTTATGTGGTCGGAAACGCTTCCTTCTACTTGTTACATATATATGTAAT

mel-CG7692      ------------------------------------------------------------
mel-CG9902      TACCAACGAATCTCGTATACCCTTCTGGTCTATAGTAAGTGTTTTGTATTCTTGTTTCTT
                         5'breakpoint
mel-CG7692      --------------- AATTACTTTTGCCTGCGAGAT-AAATTTAAGTTGGCAACAATGGC
mel-CG9902      TCAAATAAATTTTTA AATTACTTTTGCCTGCATAATAAAATTTCAGTTGGCAACAATGGC

mel-CG7692      CCTAGCGAGTTCACCTTCCCCATTCAGCAATTACAGCTAGTCTCGTGGTTTAGCGAGTAC
mel-CG9902      CTTAGCGAATGCACCTTCCACATTTAGCAAATACGCCTAGCCTTACAGTTTAGCGAGTAT

mel-CG7692      ATGAACCTCG---AGATAGCGCTGCCACTAT-GTTTAAAACCATCCACTTACGGTAATCG
mel-CG9902      ATGAACCTCGTGTAGACAGCGTTGCCCCTTTCGATTAA---CATCGATTTACAGTAATCG
                                              Transcription Start Position
mel-CG7692      GCGTCAGCTGTTG-------------- CAAGCTAAATAAAACTAAGTACAAGCCGGCTGA
mel-CG9902      GGTTCAGCTGTTGTTCGTTGCAACTTT CAAGCTAAATTAAAATAAATCGAAACCGGCAAA
                                                                   Start Codon
mel-CG7692      AAAAC—AAATCGACTTTTCGCAGGATTGTCATGAATTAGGTACTGCGGA ATG GATCTAA
mel-CG9902      AAAAAGGAAATCGACTTGACATAGCATTGCCATGAATTAAGTGCTGCAAA ATG GATATAA

mel-CG7692      TATTTTCTCAGGATGTGCACGATGCAGAAAAGTTCCTGGATTGTGCGGAGAAACTGGAGT
mel-CG9902      TATTTTCTCAGGATGTGCACGATGAACAAAAGTTTCTGGATTGTGCGAGGAAACTAGAGT
                          Exon1
mel-CG7692      ACGATGAGTTCTCGGCG GTAAGGACACGCTTTGGACATATATCCTGAACTCCCTGTTTTA
mel-CG9902      ACGACGAGTTTTCGGCG GTAAGGACTTGCTTTGTACATAAATCCTTATGCACACGTTTTC
```

```
                                          Exon2
mel-CG7692      ATGGCTTAAATCTTGA-TCTACAG AAAATGTTTGTATTCTGCAATGCGCACAGCGATGTG
mel-CG9902      ATTGCTTAAATCTTGGTTTTATAG AAAATGTTTGTATTCTGCAATAAGCACAGCGCTTTG

mel-CG7692      GATGTGTTTGAGCTAATGGGCAATGACCGGTTGTCGCAGTTAATCTACGGATGGGAGGTG
mel-CG9902      GATGTGTTTGAGCTAATGGGCGATGACCGGTTGTCGCAGTTACTCTACGGATGGGAGGTG

mel-CG7692      TGCCATCTGCCCACCAGGGATTCCGAGTCCTTAAAGGACCCAGCGCTGCGTCACATGGTG
mel-CG9902      TGCCACCTGCCTACCAGGGATTCCGAGTCGCTAAAGGACCCAGCGCTGCGTCACATGGTC

mel-CG7692      CGGGTTTTAGCGCACCACTCGCTGCTGATTTGGTTTAGCCAGGAGTGGAAGACCTGCTCC
mel-CG9902      AGGGTTTTGGTCTACCACTCGCTGCTGATGTGGTTTAGCCAAGAGTGGAAGACCTGCTCC

mel-CG7692      GAAGGCCTTAAGCAACTATTGCTCTACAGCCTCACCAAAGCGGTGCCGTCGTTCTACCAG
mel-CG9902      GAAGGCCTCAAGCAGCTTTTGCTCTACACCCTCACCAAAGCAATGCCGGCATTCCATCAG

mel-CG7692      ATTGGTGAACTGGCCAATTTCAACTGGTCGTACGTTCTGCAGTTCCAGACAAGGCCCTGG
mel-CG9902      ATTGGTGAACTGGCCAATATCAACTGGTCGTACGTTCTGCAGTTCCAGACGAAGCCCTGG

mel-CG7692      AGCCTGCCCTACCTGCAGCAGCTATTCCTTAACCTGAAGCTGAACAAACACCACGCCATC
mel-CG9902      AGCCTGCCCTACCTGCATCAGCTATTTCTTAACCTGGAGCTGAATAAACAACACGGCATC
                                      Exon2
mel-CG7692      GAGCACCCAGTTCCTGCGGCATCAGGAGAAG GCAAGCAATTACTATTTTTCCCGAAGGAT
mel-CG9902      GAACACAAAGTTCAAGCGGCATCAGGAGAAG GTAAGCCATTACTAATTTTCGTCAAGGAT
                                           Exon3
mel-CG7692      TTATCTAATCATAGTC-AATTTCTTAG AAGTTGCTTTCCTGATACAGGAAGGGATATCTC
mel-CG9902      TCATCTAATCATTGCCTTATTTCTTAG ATGTTGCTTTCCTCATGCAGGAAGGGGTATCTC

mel-CG7692      TGGCCCTGCTGCGGCTAATACAGCTCTTTAATGCCGGCAACTTTGAGGCCGTGAAACAGC
mel-CG9902      TGGCCCTGCTGCGACTAATCCAGCTCTTTAACGCCGGCAACTTTGAGGCTGTAAAACAGC

mel-CG7692      TGGCGCTTCGGATGCTGGCTGCTTGGGTGAAAGCACACGAAAATGATCAATTTGTGTCCT
mel-CG9902      TGGCGCTTCGGATGCTAGCTGCTTGGCTGAAAATCCACGAAAATGCACCATTTGTGTCCT

mel-CG7692      TTGAAGGGGATAAAAACTCAATTACACTAATCGGTCATCTATACCTCTTGACTGTTTTTG
mel-CG9902      TTAAAGGGGATGAAAACACAATTACACTAATCGGTCATGTATGCCTCTTGGCCGCTTTTG
                                            Exon3
mel-CG7692      TAAGAGATGAAAATCGAGGCTTTGTAATAGATAATTTG GTAAGGAAAACATACATGATA-
mel-CG9902      TTAGGGATGATAATCAAGGCTTTGTGATTGATAATTTG GTAAGGAAAACATTCATCATGT
                                                            Exon4
mel-CG7692      ---CAACATATC-AAACACAATGTCATCATAAAACCACTCCTTAG ATGTACAACATACGG
mel-CG9902      AACTAACATAACAAAACTAAATGTCGTCATAAAACCACCTCGTAG ATGTACAACATAGGG

mel-CG7692      TTCTATACCCAGATCATGCAGGAAGCTTCGATGTTACATGACATTAACTTTACGCCAGCT
mel-CG9902      TTCTATGTCCAGATCATGGAGGAATTTTCGAGCTCACATGATATTAACTTTACGCCAGCT

mel-CG7692      CGGCTTATTGCCCACGTTTGCGTGCGTCTTCAACTCGGAAAAAATGGGTTCTTTGAGCAA
mel-CG9902      CGGCTTATTGCCCGCGTTTGCGTCCGTCTTCGACTCGGAAAGAATGCGTTTTTTGAGAAA

mel-CG7692      ATTGGGTTTAGGAAATTCAAATTCAGTCTGGTCACTTCATTCGCCGTCATGCTGGAGGTC
mel-CG9902      ATTGCATTTAGGAAATTCAATCTATGTATGGTCACTTCATTTGCCGTCATACTAGATGCC

mel-CG7692      TACGCCAGCTATGTGCTTGGGAATCTCCTGATGGAGCTCCAGGCGCTCAACGAACACGAT
mel-CG9902      TACTCCAGCTATGTGCTGGGAAATTTATTGATGGATCTTCAGTCGCTTAACGAGCACGAT

mel-CG7692      TTTCTGGAGCACCTGCCACAGTTCAAAGAGCTAATGTATCGCTATGTAGAGGAGCGAGAG
mel-CG9902      TTCCTGGAGCACCTGCCACAGTTCAAAAAGCTAATGGATCGCTATGTAGAGGAGCGCGAG

mel-CG7692      AGCAGCGAGCGCTTCCTTCTAGAGGAACTCAAGAAGAAGGAAAACCAGCGAAACAGCCAC
mel-CG9902      AGCAGCGAGCGCTTTCTTCTAAACGAGCTCAAGAAGATGGAACAACAGCGAAACAGCTGC

mel-CG7692      TTTCAACCACATGTAGTAGAGCTAAATCTCAACTACCAGCAGCAGATATGCAAGGGAAAC
mel-CG9902      ATTGAACCGCATCTAGTAAACCTAATTCTCAACTACAAGCAGCAGATATGCAAGGGAAAT

mel-CG7692      CGAGCCAGCAACATAGGCGACTACAAGAACTGTGATGACGACGAACAGATTCAGCCTGAG
mel-CG9902      CGAGCCAGCAACATAAGCAACTACAAGAACAGTGATGACGACGAAAAGATTCAGCCTGAG

mel-CG7692      GTGGACGAAGAACAAGATAGACAAATAGATCCGGTGTTTCAAAATGTACCAAACAATGAG
mel-CG9902      GTGGGCGAAGAACAAGATAGACAAGTAGATCCGGTTTTTCAAAATGTACCAAACAATGAG
                                              Exon4
mel-CG7692      GAAGTTTTGAGCTTTGTGTACAAACTGCTGGCAGAGCGG GTAAGTGATGAGAACTATGAA
mel-CG9902      GAAGTTTTGAGCTTTGTGTACAAATTGCTGGCAGAGCGG GTAAGTGATGAGAACTAACAA
```

```
                                                        Exon5
mel-CG7692      AAGGAATTCCTGTACTAAACACAGTGCTTTC---ATGTAG AATTTTTCAGGATGGACATT
mel-CG9902      AAGCGAAAACTTTACTAAGCACCGCGCTTTTTAATTTAG AATTTTTCAGGCTGGAGATT

mel-CG7692      TGCTAAGATTGCCCTGCTGCTGAAGATCATTGGACAACAGTTGAATGCAATTGAAATATG
mel-CG9902      TGCTAAGATTGCTCTGCTGCTGAAAATTATTGGGCAACAAATAAATGCATTAGAAATGTG

mel-CG7692      GAAATATCATCCCGGATTAACAGTTGATTTTATGCTTAATTTGGAGCAAAAGATGTCGGA
mel-CG9902      GAGATATCATCCCGGGTTAACCGTTGATTTTATGCTCGATTTGGAGCAAAAGATGTCTGC

mel-CG7692      AAACTATGCTGACCTAGCCAAGGTATTCTCCGAACATGGGTTTATGGAGGCAGAATTCTG
mel-CG9902      AAACTATGCTGACCTAGCCAAATTGTTCTCTGACCATGGATTTATGGAGGCAGAATTCTG

mel-CG7692      GCTTACGGCTTTCTACCTCCACCCCACATCATCAAATTATAACGAAGTGAGGCGCTGTTC
mel-CG9902      GCTGACTGCTTTCTACCTCCACCCCACATCATCAAATTATAACGAAGTAAAGCGCTGTTC

mel-CG7692      GCGTATTAAGAAAAAACGGCAGGAGGACGACTTGCTACCTGCTACGACACCACAGAACGT
mel-CG9902      GTGGATCATGAAAAATCGACAGGAGGAGGGCGGGCTCCCTGCTCCAGCGATTGGCAACGT

mel-CG7692      CAAGTACGAACTGCTGAGCTCCACCATCGATGTGGACGAGATTGTAAAAATAACGAACCA
mel-CG9902      CAAGTACGAACTGCTGAGCTCCACCATCGATGTGCACCAGATTATGACCATAACAAACCA

mel-CG7692      TTACGATCCAGTGGCCGACTACGATCCGATACACAAATCGCTGCAGGCGTTGCGGCTTCC
mel-CG9902      TGAAGATCCAGTGTCCGACTACGATCCAATAAACGAGTCGCTGAAGGCGTTGCGTCTACC

mel-CG7692      CCGCAGCATGATCCAAGACCTTCTGACCGTGGTCTTCCAACCACGGAATAAACGATACTC
mel-CG9902      CAGCAACATGACCAAGGATCTTCTGACCGTGGTCTTCCAACCGCGACATAAACAATACTC

mel-CG7692      ATGGGCTCTCGATTGGCACACGCTACACGAGCGTTGCCATGCCCTTCTCAAAAGCCCGGA
mel-CG9902      ATGGGCTCTCGATTGGCAAACGCTAGACGAGCGTTGCAGTGCCCTACTCAAAAGCCAGGA

mel-CG7692      CCTCAAGAGAAAATTTGTGTCTCTTAACATGGCCGAAGCTGGCGACGATTTGAAGTACCT
mel-CG9902      CCTGAAGAGAAAATTTGTGTCTCTTAACATGGCCGAATCATGCGACGACTTGAAGTATCT

mel-CG7692      CCAGATCGACTATGCCAAATACAGGGATCGCCCTCAGTTGGACTATGGCACCATAGAAGA
mel-CG9902      CCAGATCGACTATGCCAAGTACAGGGATCGACCACAGTTGGACTATGGCACCATAGAAGA

mel-CG7692      GGGCTACGAAAATGCGGTTAATCTCCCAGAGGCTGAGGAAGAAGCTGAACAAACGTCAGC
mel-CG9902      GGGCTACGAAAATGAGACTAATCTCTCGAATGCAGCTGGAAAGGCTGAATCAACACCAGC

mel-CG7692      AGTGGATGATGAAGCAGAGAAAGCCAAGGAGCAGAAAAAGCGGCGACCCGCAAAACGCAA
mel-CG9902      AGTGCATGATAAAGCAGAGAAAGCCAAGGAGCTGAAAATGCGGCGACACGCAAAACGCAA
                      Exon5
mel-CG7692      ATTTTGGGACGAAG GTTAGCAACAAAATCGTTTTCTTTTGAGCCACTTCCTTTCATAATA
mel-CG9902      ATGTTGGTATGAAG GTTAGCAACGAAATCTTTTTTCTTTGAGCAACTTTCATATAAGATT

mel-CG7692      ACTAATAATTCAGTCATACTAATAATTTCAATTTAAAATCCC------------------
mel-CG9902      A-----GATTCAGTCAAACTAATGAATTGAATTTTAAATCCCACAGTTTCGGAAGACGAG

mel-CG7692      ------------------------------------------------------------
mel-CG9902      GAAGAGGCTGCCTCAAGTGATTCGGAGCCCAGCTACACAGGAAATGGCCGCCGAACACAG

mel-CG7692      ------------------------------------------------------------
mel-CG9902      ATTAGAGCCGCAGCCATTGCATCCAAAGCTAATGGTCGCCGGACTGGGATTAGAGCCGCA

mel-CG7692      ------------------------------------------------------------
mel-CG9902      GCCATTGCAGCCAATATCAAATTGTCTGAAATGTCCTTTCCAGAGACATCGAATAGGCGC

mel-CG7692      ------------------------------------------------------------
mel-CG9902      TTTTCTACACAGCCCGTCGTCAAGGAACAGCCAAAAATCGCCGTCCAGCCTAAAAAACAA
                        Exon6
mel-CG7692      ---ACAG TAGTATTTTCGGAAGACGAGGAGGAGGCTGCCTCAAGCGATTCGGAGCCCTAC
mel-CG9902      AAAACAG TATTGTTTTCGAAAGAAGAGGATGAGGTTTCCTCAAGTGATTCGGAGCCCTGT

mel-CG7692      TACACAGGAAATGGCCGCCGGACTCGGGTTAGGGCGGCAGCCATGGTAGCCAATGCCATG
mel-CG9902      TACACAGGAAATGGCCGCCGGACTCAGATGAGAGCCGCAGCCATTGCAGCCAAGGCCAAA

mel-CG7692      TTGTCTGACA--------------------------------------------------
mel-CG9902      TTGTCTGAAATCTCCTCTCCAGAGACATCGAATAAGCACTTTTCTACACGGTCCATCGTC

mel-CG7692      ------------------------------------------------------------
mel-CG9902      AACGAGCAGCCAAGAATCGCAGTCCAGTCTCGAAAACAAAAAGCAGTAATGTTTTCGAAA
```

```
mel-CG7692      -----------------------------------------------------------------
mel-CG9902      GACGGGGATGAGACTTCCTCAAGCGATTCGGAGCACTGCTACACCAGAAATGGCCACCTG

mel-CG7692      -----------------------------------------------------TGGATCGAAGT
mel-CG9902      AATCGGATTAGAGCCGCAGCCATTGCAGCCAAAGCCAATTTGCCTGAATTGGATCGAAAA

mel-CG7692      GTGCGCGGTGGGCGACGATCGAGCTCTCCAGAGACATCACATCACCAATTTCATACGCAG
mel-CG9902      GTGAGCGGTGGGCGACGATCGATCTTTCCAGAGGCATCGGGTAATCACTTTGTTACACAG

mel-CG7692      CCCGTCGTCGAGGAGCAGCCAAAAATCGCAGTCCAGCCTCCAAAACAACTGTGTCTACAA
mel-CG9902      CCCGTCGTCGCGGAGCAGCCAAAAATCGCAGTTCAGCCGCAAAATCAAC-----------

mel-CG7692      GAAACATCTGCGCGTGTCGAAAAGCGTACGATTAGCGAACTGATGGAGTCACGACCCAAA
mel-CG9902      --------------------AAAAGCGTACAATTGCCGAGCTGATGAAGTCACGACCCATG

mel-CG7692      TTTACCAACGAAACCGTCGGTTTTAAGCCGATCGCCGACATATGGAGTTTCAAGAAGGAA
mel-CG9902      TTTACCAACGAAACGAAAGGATTTAAGCCGATCGCCGACATATGGAGTTTCAGCAAGGAA

mel-CG7692      GAGCTGCAGAATGTGGTAGAAGACAGCAGCAGCATGATAGAGTGCAGCTCGATATTAAAC
mel-CG9902      GAGCTAGAGAATGTGGTTAGCGGGCCCAACGACATGATAGAGTGCAGCTCGATTATAGCC

mel-CG7692      AAATTCAAAATCTTGAAGGCCCTCAAAATGGCCAAAGCTGCAAAAGCCGCCGTGCAGCCA
mel-CG9902      AAAATGAAGTTATTGAAGGACCTCAAAATGGCGAAATCTGCAAAAGCAGCCGGACAGCCA

mel-CG7692      TGTTCCCAGCTTGTTCAAAATATCGTTCGCACGGGATCGGAAACTGAAAGCGTAAACTCA
mel-CG9902      TGTTCACAGC------------TCGTTCGCACAGGATCAGAAACTGAAAGCGTGAATTCC

mel-CG7692      GAAACTTCCACGATGGCTACCCACGACTTCAACGAGGAGGAGGTCAGCGAAGGTTCTACC
mel-CG9902      GAAACTTCCACGATGGCTACACACGAATTTAACGAGAAGGGGACCACTGTAGGTTCCCCC

mel-CG7692      GGTGTAAGTAAAGAGC---TAACACCTTCGCCGACCACAAAGTCTGATTTAACCATGGAC
mel-CG9902      GGTGAAGGTAAAGAGCAAAAAACACCTTCGCCGACCGCAAACTCCGAATTAAGCACTGAA

mel-CG7692      CCAGAAGTGTTGCAGCCCACTGGTGAGACCAAGACGCATTTGATCCATGGGAGCCTAGAA
mel-CG9902      ACAGAAGTGTTGAAGCCCACCGATGAGACTGAAGCGCATATGATCCATGACGCCCTAAAA

mel-CG7692      ACTACAAAAGTGCCACAGAAGACTCCGGAGTTTCTGTTTTTGACCAAGGAGCCCGTGAGA
mel-CG9902      ACTACAAACGTGCCACAGAAGACTCTGGACTTACAGTTCTTGTCCAAGAAGCACATAAGA

mel-CG7692      ACTGGACAACAGACCCAGGAGCCACTGGGAGATGCCTTAAAAACTAGTGACCTAAAACAG
mel-CG9902      ACTGGATAACAGACCCAGGAGTCCCCAGGAGATGTCATAGAAACTAGTGATCTCAAACAG

mel-CG7692      GTCATCCCAAATGGACTGTTCAAAGTAGAGGGACTGGAAGCGAGACAGGCGGCCGAAAAG
mel-CG9902      GTCCCCCTAAATAGCCTGTTCAAAGTAGAGGGACTGGATGCAAGACAGGCGACAGGAAAG

mel-CG7692      CCAAAACAAACTGGACAACCGCAACGGGATCTACCGAATACAAGAAAACAAAAAGATAAG
mel-CG9902      CCAAAAAAAAAGGAACAAACGGAACAGGATCTGCCAAATACTAGAAAACACAAAGATAAG
           3'breakpoint
mel-CG7692      CTT
mel-CG9902      CTT
```

10. CG2952/CG2952-r

```
         5'breakpoint    Start Codon
yak-CG2952        AAA ATG GCCGACAAGAAGAATCTCCTCCTGCTGTTCGAGCATCCCACGGAGCCCGTCTTC
yak-CG2952-r      AAA ATG GGCGACAAGAAGAATCTCCTCCTTCTGTTCGATCACCCGACGGAGCCCGTTTTC

yak-CG2952        ATGGACAAGGGCGGCAACGGCACCTTGTTCGATGTGCCCGCCTCCTACGTGACCGAGCGC
yak-CG2952-r      ATGGACAAAGGTGGCGATGGCACCATGTTCAATGTACCCGACCACTATCTGACCGATCAA

yak-CG2952        TACAGCAAGATGTGCAGAAACGTCCAGCGGCGCGTTAGCGGTGG--GTTCGAGAAGTGCG
yak-CG2952-r      TACACGAAGATCTGCAAGAAGACGCAGTACCGAATTGGCGGTAGTAAAGCGAGAATGATA

yak-CG2952        TCCTGGTCAAGGAGATCGAGATCCCAGATCTCAGCTGCCCCATGAGACTGGGCCGTTCGG
yak-CG2952-r      GACC--TCAAGGAGATCTCCATTCCCGATCTTAGCTTTCCCATGTCCTTGGGCCGTACCG

yak-CG2952        AGCAGTTCTCGCACTTCCTGAAGTCCCACCGCCAGATGGCCAGCTCTTTGATCGATGTCT
yak-CG2952-r      AGCAGTTCTCGCTGATGCTGAAATCCCACCGCCAGATGGCGGGGCACTTGATCGAGGTCT

yak-CG2952        TCATCAAAATGCCCACCGTAGATGAACTGCAGAGCGTGGCGGTGTACGCCAGAGATCGTG
yak-CG2952-r      TTACCAAACTGCTTTCCGTGGAGGATCTCCTAAGTGTGGCCGCCTTTGCCAGGGATCGCG
```

```
yak-CG2952        TCAATCCGGTGCTCTTCAACTACGCCTTGTCGGTGGCCATGCTCCATCGTCCGGACACCA
yak-CG2952-r      TTAACGCTGTAATGTTTCATTATGCCCTATCGGTGGCTCTGCTCCATCGTCCTGACACCC

yak-CG2952        AGGACCTGGGTCTGCCCACCTTCGCGGAGATCTTCCCAGATCACTTCATCGACTCGCAGA
yak-CG2952-r      AGGGCCTAGATCTGCCATCCTTTGCGCAAAGCTTTCCAGATCGATTCATCGACTCGCAGG

yak-CG2952        TGATTCGCAATATGCGCGAGGAGTCCTTTGTGGTGGAGCAGACCGCTGCCCGCCTGCCCG
yak-CG2952-r      TACTGCGCTCGGTACGCGAGCAATCGTTCGTGGTGGAGGAATCGGAATCACGGGTTCCTA

yak-CG2952        TCGTTAATTCGGTCAAGTATACCGCCTCTGACTTCGACGTGGAGCACCGGCTGTGGTACT
yak-CG2952-r      TTGTTGTTCCAGTTAACTATACCGCCTCCGATTTGGATCACGAGCACCGTCTGTGGTACT

yak-CG2952        TCCGCGAGGACTTGGGCGTCAACCTGCACCACTGGCACTGGCATCTGGTCTATCCCAT--
yak-CG2952-r      TTCGCGAGGATCTGGGCATCAATCTGCACCACTGGCACTGGCATCTCATCTATCCCATCG

yak-CG2952        -------AGATGCCCCCGACCGCAGCATCGTGGACAAGGACCGCCGTGGCGAGCTGTTCT
yak-CG2952-r      AGATCAGCGATGGTGCCGATCGCCGGATAGTGGACAAGGATCGTCGTGGCGAGCTTTTCT

yak-CG2952        ACTACATGCACCAGCAGATCGTTGCCCGCTACAATGCCGAGCGGCTGAGCAACCACATGG
yak-CG2952-r      ACTATATGCACCAGCAGGTGATGGCCCGCTACAACGCCGAGCGATTGAGCAATCACATGG

yak-CG2952        CCCGGGTGCAGCCGTTCAACAACCTAGACGAGCCCATTGCCGAGGGCTACTTCCCCAAGA
yak-CG2952-r      CCCGGGTTCAGCCGCTTAACGACTTGGATGAACCCATTGCCGAAGGCTACTTCCCCAAGA

yak-CG2952        TGGACTCCATGGTGGCCAGCAGGGCGTATCCTCCGCGCTTCGACAACACCCGGCTGAGCG
yak-CG2952-r      TGGACTCCACGGTGGCCAGTCGGGCCTATCCTCCGCGATTCGATAACACCCAGCTGAGCG

yak-CG2952        ACGTAGATCGCCCCAACAACCAGTTGAGAGTGGGAATCGACGACATGAAGCGATGGCGCG
yak-CG2952-r      ATGTCGACCGCCCGGCCAACCAGATCAAAGTGGGGCTCAACGACATGAAGCGGTGGATCG
                                                         Exon1
yak-CG2952        AGCGCATCTACGAGGCCATCCACCAGGGCTACGTTTTTGAT GTGAGT-GCAGCTCTAGCT
yak-CG2952-r      ATCGCATCTACCAAGCCATCCACCAGGGCTACGTTTTGGAT GTGAGTCTTGGCCATTGTC
                                                                          Exon2
yak-CG2952        CTCTGAATGCCGGAATATAT----ACATATAGTTGGT-TCGTTGT—AG GATAACAATGA
yak-CG2952-r      CTTTCAAT-------TATTTTTCATCAAGTACCTAATCTCATTTTCCAG ACAAACAATGA

yak-CG2952        AAAGATTGCCCTGGATGACGTGAAGGGGATCGACATCCTGGGCAACATCATAGAGGCCTC
yak-CG2952-r      AAGGATTCAGTTGGATGAAGTGAAGGGCATTGACTACCTCGGCAACATTATTGAAGCCTC
                         Exon2
yak-CG2952        CGCATTGACGCCCAACA GTACTCTGGTAAGCGATTTAGTTGCATAGACACATAGCATAAT
yak-CG2952-r      CGTATTGTCGCCCAATA CAGCACTGGTAAGA---TGAGCTACTTTGCCA-----------
                                                                       Exon3
yak-CG2952        GGTAATCTCTTGAGCAACAAATTCCTAAATTTCATCTCATCTGCAG TACGGCGACTTGCA
yak-CG2952-r      ------CTCCTATGCA--TAGTTACTAGAAT—ATCTAATCTGCAG TACGGTGATGTGCA

yak-CG2952        CAACAAGGGGCACATGCTGATTGCGTACTCCCACGACCCAATCAACAAGCACCTGGAGTA
yak-CG2952-r      CAACATGGGTCACGTCTTGATCTCGTACTCCCACGATCCGACTAACAAGCATTTGGAGAA

yak-CG2952        TGCGGGCGTGATGGGCGACTCCTCCACCGCGATGCGCGATCCGATCTTCTACAAGTGGCA
yak-CG2952-r      CCAGGGTGTGATGGGGGATTCTACCACTGCGATGCGCGACCCAGTCTTCTACAAGTGGCA

yak-CG2952        CGCCTTCATAGACAACATTTTCCAGGAGCACAAGCGTCAGCTTACCCCCTATGAGAAGAA
yak-CG2952-r      CGCGTTCATTGACAACATTTTCCAGGAGCACAAGGGGCGACTTCCGGCCTACACCGAGGA

yak-CG2952        GGACCTAAGCTTTCCGGACGTGCGCGTCCAGAGTATTGAAGTGGAGAGCCAGGGTAAGAT
yak-CG2952-r      AGACCTGAGCTATCCCGGCATTACGGTTCAAAACATCAATGTGATTAGCCAGGATAGGAT

yak-CG2952        CAACAGGCTGACCACATTCTGGCAGGAGTCCGATGTGGACATGTCCCGCGGCCTTGATTT
yak-CG2952-r      CAACAGGATAACCACATTCTGGCAGGAGTCGGATGTGGATATGTCCCGTGGCGTCGACTT

yak-CG2952        CGTGCCGCGCGGCCACGTCCTCGCTCGGTTCACCCATCTGCAGCACCACCCATTCAGCTA
yak-CG2952-r      TTTGCCCCGTGGCAGTGTCGTTGCTCGATTCACCCATCTGCAGCACAACCCATTCACCTA

yak-CG2952        CACCATCAAGGTGGAGAACTCCAGCGAGGCCACGCAGTATGGATACGTGCGCATCTTCAT
yak-CG2952-r      CTCCATTAACATGGTCAACTCCACTGGAGCCACCAAGTTCGGCTACGTGCGCATCTTCAT

yak-CG2952        TGCGCCCAAGATGGATGATCGTAACAATCCCATGCCGCTGGAGGAGCAGCGTCTGATGAT
yak-CG2952-r      GGCGCCCAAGCTGGATGATCGTAACGCTCCCATGTTTATGGCAGATCAGCGCCGCATGAT

yak-CG2952        GGTGGAGATGGACAAGTTCGTTGTTACCATGCCACCCGGAAGCCAAACTATTACCCGGAA
yak-CG2952-r      GATTGAGCTGGACAAGTTTGGGATTTCGATTCCACCCGGACAACATAGGTTTGTACGCAG
```

```
yak-CG2952          CTCCACGGAGTCGAGTGTCACCATTCCGTTTGAGCGCACCTTCCGGAACCTGGACCA---
yak-CG2952-r        CTCTACGGAATCGAGTGTAACCATTCCCTTTAAGCGCACTTTCCGCAATCTGGACAACAA

yak-CG2952          ------------------GCTGGAGGAGCTGGAGAATTTCATGTGCGGCTGCGGCTGGCC
yak-CG2952-r        CCGTGGGGCTGCGAATTCTCCGGAGGAGTTGGAGAACTTTTTCTGCGGCTGCGGTTGGCC

yak-CG2952          CCAGCACATGTTGATTCCGAAAGGTCGGGCCGAGGGTCTGAGTTTCGAGCTCTTCGTCAT
yak-CG2952-r        CAATCACATGTTGGTCCCAAAGGGTCGTGTTGAGGGCATGAGCTTTGAGTTGTTCGTCAT
                                      Exon3
yak-CG2952          GGTTTCCAACTACGAAGACGATAAG GTAAGGTGTCCTTAAAGTATACTGCTGCTGCCTCC
yak-CG2952-r        GATTTCCAACTACGACAATGAAAGG GT-AGGTGTTCTCTAAGGGCTTTATGGACTTGTCC
                                                  Exon4
yak-CG2952          TGATCCCAACCCTCTTAACTCCTGCAG GTGGAACAGAAGGCAGCGGATTGTGCCTGCAGC
yak-CG2952-r        TGA-------CGTTTCACCTTCTCCAG GTTGACCAGACACTTGTGGGTAG---CTGCAGC

yak-CG2952          ATTGCCGCTTCCTACTGCGGACTGCGCGATCGCCTCTATCCGGACCGCAAGTCCATGGGC
yak-CG2952-r        GATGCCGCCTCCTACTGTGGAGTCCGCGATCGCCTTTACCCAGATCGCCGATCCATGGGC

yak-CG2952          TATCCCTTCGATCGCAGCATCCGCCGGGGTTCTGAGATGTTGGATAGGTTCCTTACGCCC
yak-CG2952-r        TTCCCATTCGATCGCGTGCCTCGCATGAGTGCAGTTTTGCTGAAGGACTTCCTAACGCCC
                                                                   3’breakpoint
yak-CG2952          AACATGCGCGCCGTCGAGGTCATCATTACCCATGAGTCCCGTACCGAAAAGCTTCCC ---
yak-CG2952-r        AACATGAGCACTGTGGAAGTCAACATTACCCACGAGGACCGCACAGTAAAGCTTCCC CAA

yak-CG2952          ------------------------------------------------------------
yak-CG2952-r        TTGGCAAACTTATCCTGATCTACATATATGTATCCATATATGTATATCTATATTATAGAT
                                        (TATATG)n Simple-repeat
yak-CG2952          ----
yak-CG2952-r        GTAT
```
