# Supplementary material for: Repetitive Element-Mediated Recombination as a Mechanism for New Gene Origination in Drosophila
Source: PLoS Genet. 2008 Jan 18;4(1):e3. doi: 10.1371/journal.pgen.0040003 (PMC2211543; doi:10.1371/journal.pgen.0040003)
Supplement: Supplementary file 2 [file pgen.0040003.sg002.pdf]

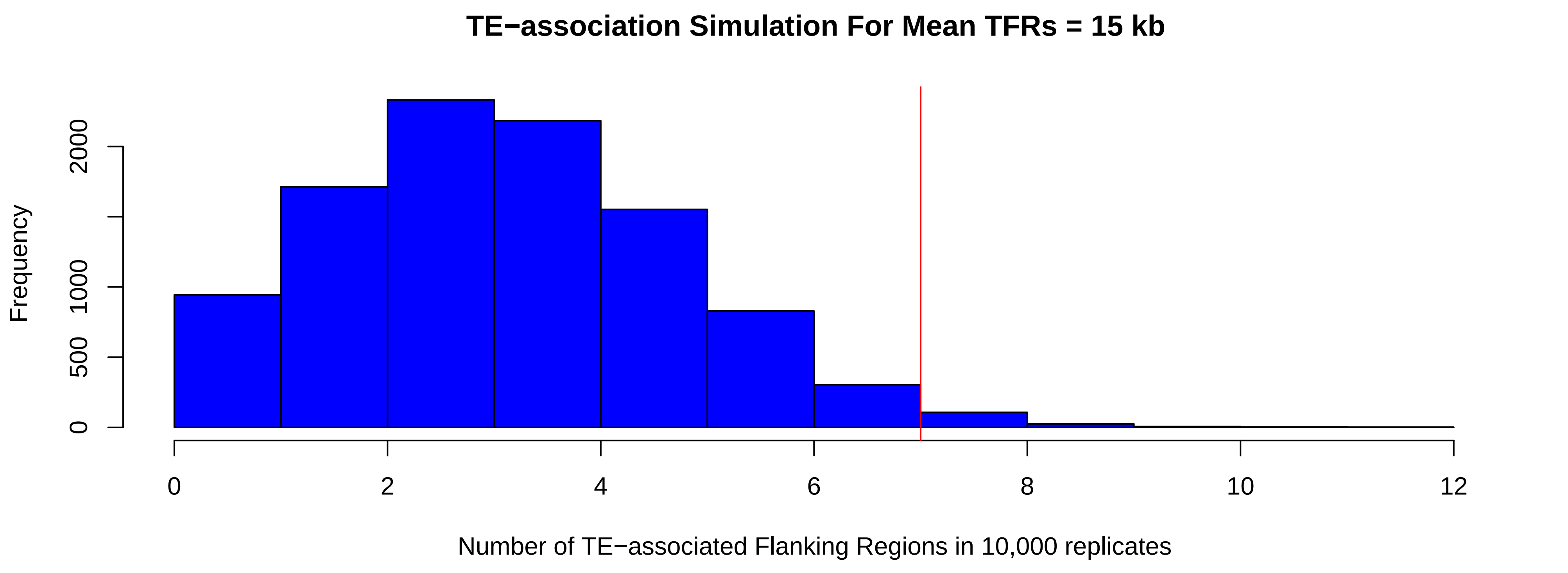
TE−association Simulation For Mean TFRs = 15 kb
Frequency
0
500
1000
2000
0
2
4
6
8
10
12
Number of TE−associated Flanking Regions in 10,000 replicates
