# Supplementary material for: Repetitive Element-Mediated Recombination as a Mechanism for New Gene Origination in Drosophila
Source: PLoS Genet. 2008 Jan 18;4(1):e3. doi: 10.1371/journal.pgen.0040003 (PMC2211543; doi:10.1371/journal.pgen.0040003)
Supplement: Supplementary file 3 [file pgen.0040003.st001.doc]

**Supplementary Table 1** Expression patterns of the new and parental genes

| Gene ID | Species Detected | Embryos | Larvae 2 | Larvae 3 | Pupae | Adults | | |
| --- | --- | --- | --- | --- | --- | --- | --- | --- |
|  |  |  |  |  |  | Male | Female | |
| kep1 | mel | + | + | + | + | + | + | |
| CG4021 |  | - | + | + | + | + | - | |
| CG3875 |  | - | + | + | + | + | - | |
| CG3927 |  | - | + | + | + | + | - | |
| CR9337-r | sch | - | + | +(weak) | + | + | +(weak) | |
|  |  |  |  |  |  |  |  | |
| CG7692 | mel | + | + | + | + | + | + | |
| CG9902 |  | +(weak) | +(weak) | + | + | - | + | |
|  |  |  |  |  |  |  |  | |
| CG8095 | mel | + | + | + | + | + | + | |
| CG5372 |  | - | - | + | +(weak) | - | - | |
|  |  |  |  |  |  |  |  | |
| Ballchen | sim  mau  sch | + | + | + | + | + | | |
| Hun |  | - | - | - | + | + | | - |
|  |  |  |  |  |  |  | | |
| CG7635 | sim | + | + | + | + | + | | |
| CG7635-r |  | + | + | + | +(weak) | +(weak) | | |
|  |  |  |  |  |  |  | | |
| CG3071 | sim | + | + | + | + | + | | |
| CG3071-r |  | + | + | + | + | - | | |
|  |  |  |  |  |  |  | | |
| CG3101 | sim | + | + | + | + | + | | |
| CG3101-r |  | +(weak) | +(weak) | + | + | + | | |
|  |  |  |  |  |  |  | | |
| CG8490 | sim | + | +(weak) | +(weak) | + | + | | |
| CG8490-r |  | - | +(weak) | +(weak) | - | - | | |
|  |  |  |  |  |  |  | | |
| klg | sim | + | + | + | + | + | | |
| klg-r |  | +(weak) | - | - | - | +(weak) | | |
|  |  |  |  |  |  |  | | |
| d | yak | + | + | + | + | + | | |
| d-r |  | - | - | +(weak) | - | - | | |
|  |  |  |  |  |  |  | | |
| Dox-A3 | yak | + | + | + | + | +(weak) | | |
| Dox-A3-r |  | - | +(weak) | +(weak) | - | - | | |

Note: + means expression， - means no expression.

Primers for the RT-PCR expression analyses.

| kep1-F | AGTTTACTGAGAAACAGCCACCC |
| --- | --- |
| kep1-R | GGTGTGCGACGAAATCAACC |
|  |  |
| CG4021-F | AGATTACTGAGGAACAGCCAACT |
| CG4021-R | TTCATAACGTAGGTGCCTATACC |
|  |  |
| CG3875-F | CCAAGTTCAACTTCGCTCGG |
| CG3875-R | TGCGACGAAATCACTTGGC |
|  |  |
| CG3927-F | CATTCCCGGCAAGGAGCC |
| CG3927-R | GTCCACATAGCAGGATTGGGC |
|  |  |
| CR9337-r-F | GAGTTAACTGAGAAAGAGCCACT |
| CR9337-r-R | ACTCCAGAGTCCGTTACTGTTC |
|  |  |
| CG7692-F | GACTTGCTACCTGCTACGACA |
| CG7692-R | TCCTCGTCTGAAAATACTA |
|  |  |
| CG9902-F | GAAGGCGTTGCGTCTACCCA |
| CG9902-R | CATTTGCGTTTTGCGTGTCG |
|  |  |
| scb-F | GATCACGGCCTGCTACAGACTGAG |
| scb-R | TGAATTGGGGCAGGTAGGCG |
|  |  |
| CG5372-F | GTCAAAATCCATGCGACTGTG |
| CG5372-R | CAAGGAGCCAAAGATGAAACTAG |
|  |  |
| Ballchen-F | AACAACGATCTTGACCAGAGG |
| Ballchen-R | TGACTTGGGTTCAGTCTTAACG |
|  |  |
| Hun-F | AACAACGATCTTGACCAGAGG |
| Hun-R | GACTTGGGTTTAGTCTGGTCTG |
|  |  |
| Mec2-F | GGCGGAGCTCGTGGTACC |
| Mec2-R | CGGCCTCCGCCTCGACT |
|  |  |
| CG7635-r-F | CGGCGGAGCTCGTGGTACA |
| CG7635-r-R | CGGCCTCCGCCTCGACG |
|  |  |
| CG3071-F | CTCCTTTCTTCCACTGAACACA |
| CG3071-R | CGAAGACATCCATGACCGTC |
|  |  |
| CG3071-r-F | CTACAAACTTCCACTGAACAAGC |
| CG3071-r-R | CCTCTAAGACATCCAAGATCCTG |
|  |  |
| CG3101-F | TGTGGTTCAAGGCACCCTTC |
| CG3101-R | GCGCGTCGTCATCACTATTAC |
|  |  |
| CG3101-r-F | CCACTGTGGTTCAAAGCACTCA |
| CG3101-r-R | CGCGCATCGTCATCACTATAAG |
|  |  |
| CG8490-F | GAGTCGGTTGCTGCAGACGA |
| CG8490-R | TGGGGAATTTCTGGCTGCTG |
|  |  |
| CG8490-r-F | GAGTCGGTTGCTGCAGATGG |
| CG8490-r-R | CGGGTAATTTCTGGCTGCAA |
|  |  |
| klg-F | AGCGACTATGCAAAGTATGTTGG |
| klg-R | GCTGGCTGCTTTTCCTGAG |
|  |  |
| klg-r-F | GCGACTATGCAAGTTAATGTGAA |
| klg-r-R | GCTGGCTGCTTTTCCTGAG |
|  |  |
| d-F | GTGCGTCAGATTCGATCGTTG |
| d-R | TGTTGAAACGCACTTAACTAATTAC |
|  |  |
| d-r-F | GAGCCATCCAGCACCGACA |
| d-r-R | AAGGTAAGCTATCCCCAAAATC |
| d-r-FI | GACCTGTAGCGATCTAATCTGG |
| d-r-RI | CAGTTCCACCATCTCATTCCTT |
|  |  |
| Dox-A3-F | AGCCGTTCAACAACCTAGAC |
| Dox-A3-R | TATGATGTTGCCCAGGATGT |
|  |  |
| Dox-A3-r-F | CCTACAGGATTAGGCACCATTA |
| Dox-A3-r-R | GGTCTATCATTCTCGCTTTACTA |
|  |  |
|  |  |
| **Note** | F: forward primer |
|  | R: reverse primer |
|  | FI: forward primer of 2nd round PCR |
|  | RI: reverse primer of 2nd round PCR |
