# Supplementary material for: Repetitive Element-Mediated Recombination as a Mechanism for New Gene Origination in Drosophila
Source: PLoS Genet. 2008 Jan 18;4(1):e3. doi: 10.1371/journal.pgen.0040003 (PMC2211543; doi:10.1371/journal.pgen.0040003)
Supplement: Supplementary file 4 [file pgen.0040003.st002.doc]

**Supplementary Table 2 Substitutions** in paralogous comparisons

| Parental gene name | New-copy name | Species used in calculation | Calculated length (bp) | *Ka* | *Ks* | *Ka/Ks* | *Ka* in domain | | *Ks* in domain | | *Ka/Ks* in domain | |
| --- | --- | --- | --- | --- | --- | --- | --- | --- | --- | --- | --- | --- |
| mkg-r | mkg-r2 | mau | 1344 | 0.00595 | 0.00856 | 0.6951  (*P*=0.35) | | 0.00782 | | 0.02376 | | 0.3291  (*P*=0.20) |
| kep1 (CG3584) | CG3875 | mel | 906 | 0.10514 | 0.16713 | 0.6291  (*P*=0.05) | | 0.06743 | | 0.23599 | | 0.2857  (*P*=0.01) (*P*=0.10) |
| kep1 (CG3584) | CG4021 | mel | 906 | 0.09169 | 0.20675 | 0.4438  (*P*<0.01)  (*P*=0.30) | | 0.04531 | | 0.22060 | | 0.2054  (*P*<0.01)  (*P*=0.03) |
| kep1 (CG3584) | CR9337 | sim | 756 | 0.07653 | 0.16097 | 0.4754  (*P*<0.01)  (*P*=0.42) | | 0.03929 | | 0.17207 | | 0.2283  (*P*=0.01)  (*P*=0.06) |
| CG3875 | CG3927 | mel | 780 | 0.04082 | 0.06396 | 0.6382  (*P*=0.17) | | 0.03023 | | 0.07492 | | 0.4035  (0.13) |
| CR9337 | CR9337-r | sim | 414 | 0.00283 | 0.02820 | 0.1004  (*P*=0.04)  (*P*=0.07) | | 0.00668 | | 0.07487 | | 0.0892  (*P*=0.05)  (P=0.07) |
| CG7692 | CG9902 | mel | 2268 | 0.10049 | 0.22899 | 0.4388  (*P*<0.01)  (*P*<0.17) | | - | | - | | - |
| scb ( CG8095) | CG5372 | mel | 2532 | 0.284435 | 1.027883 | 0.2767  (*P*<0.01)  (*P*<0.01) | | 0.10839 | | 1.32806 | | 0.0816  (*P*<0.01)  (*P*<0.01) |
| Ball  (CG6386) | Hun | sim | 1365 | 0.033307 | 0.077762 | 0.4283  (*P*<0.01)  (*P*=0.29) | | 0.01592 | | 0.08033 | | 0.1982  (*P*<0.01)  (*P*=0.02) |
| Mec2 (CG7635) | CG7635-r | sim | 882 | 0.001384 | 0.012027 | 0.0156  (*P*=0.08) | | 0 | | 0.01169 | | 0  (*P*=0.10) |
| CG3071 | CG3071-r | sim | 1485 | 0.100937 | 0.092675 | 1.0892  (*P*=0.35) | | 0.06442 | | 0.10976 | | 0.5869  (*P*=0.10) |
| CG3101 | CG3101-r | sim | 960 | 0.08806 | 0.09664 | 0.9112  (*P*=0.37) | | 0.07942 | | 0.07196 | | 1.1036  (*P*=0.40) |
| CG8490 | CG8490-r | sim | 558 | 0.154124 | 0.104728 | 1.4717  (*P*=0.10) | | - | | - | | - |
| d (CG10595) | d-r | yak | 1128 | 0.043337 | 0.120097 | 0.3608  (*P*<0.01)  (*P*=0.10) | | 0.02193 | | 0.11266 | | 0.1947  (*P*<0.01)  (*P*=0.03) |
| Dox-A3 (CG2952) | Dox-A3-r | yak | 2037 | 0.186902 | 0.660535 | 0.2830  (*P*<0.01)  (*P*<0.01) | | 0.13005 | | 0.63508 | | 0.2048  (*P*<0.01)  (*P*<0.01) |
| klg ( CG6669) | klg-r | sim | 225 | 0 | 0.01297 | 0  (*P*=0.16) | | - | | - | | - |
