# Supplementary material for: Repetitive Element-Mediated Recombination as a Mechanism for New Gene Origination in Drosophila
Source: PLoS Genet. 2008 Jan 18;4(1):e3. doi: 10.1371/journal.pgen.0040003 (PMC2211543; doi:10.1371/journal.pgen.0040003)
Supplement: Supplementary file 5 [file pgen.0040003.st003.doc]

**Supplementary Table 3: Substitutions of new gene** in orthologous comparison

| New gene name | Species used in calculation | Calculated length (bp) | *Ka* | *Ks* | *Ka/Ks* | *Ka* in domain | *Ks* in domain | *Ka/Ks* in domain |
| --- | --- | --- | --- | --- | --- | --- | --- | --- |
| CG4021 | mel, sim | 948 | 0.08891 | 0.17225 | 0.5162  (*P*=0.01) | 0.03917 | 0.23320 | 0.1703  (*P*<0.01) |
| CG3875 | mel, sim | 1005 | 0.12136 | 0.13842 | 0.8767  (*P*=0.29) | 0.07573 | 0.18911 | 0.4005  (*P*=0.04) |
| CG3927 | mel, sec | 771 | 0.11097 | 0.13210 | 0.8400  ( *P*=0.27) | 0.06168 | 0.18801 | 0.3281  (*P*=0.03) |
| CR9337 | sim, sec | 915 | 0.02068 | 0.02892 | 0.7151  (*P*=0.29) | 0.00384 | 0.00946 | 0.4059  (*P*=0.29) |
| CG9902 | mel, sim | 873 | 0.065019 | 0.111714 | 0.5820  (*P*=0.04) | - | - | - |
| CG5372 | mel, sim | 3024 | 0.133701 | 0.155109 | 0.8620  (*P*=0.13) | 0.05397 | 0.12870 | 0.4193  (*P*=0.06) |
| Hun | sim, sec | 612 | 0.018822 | 0.080992 | 0.2093  (*P*=0.01) | 0.01772 | 0.09984 | 0.1775  (*P*<0.01) |
| CG7635-r | sim, sec | 606 | 0.004447 | 0.030136 | 0.14733  (*P*=0.04) | 0.00544 | 0.03014 | 0.1805  (*P*=0.05) |
| CG3071-r | sim, sec | 750 | 0.025751 | 0.011149 | 2.3091  (*P*=0.10) | 0.02601 | 0.01322 | 1.9674  (*P*=0.16) |
| CG8490-r | sim, sec | 549 | 0.019411 | 0.015872 | 1.2230  (*P*=0.38) | - | - | - |
